# Effect of feeding a gestation diet to sows for 5 days post-farrowing and feeding a liquid mixture of milk replacer and starter diet to suckling piglets on growth, selected health parameters and faecal microbiota of suckling pigs on two research farms

**DOI:** 10.1093/tas/txaf138

**Published:** 2025-10-09

**Authors:** Shiv R Vasa, Marion Girard, Gillian E Gardiner, Paul Cormican, Giuseppe Bee, Keelin O’Driscoll, Peadar G Lawlor

**Affiliations:** Teagasc, Pig Development Department, Animal and Grassland Research and Innovation Centre, Moorepark, Fermoy, Co. Cork, P61 C996, Ireland; Eco-Innovation Research Centre, Department of Science, South East Technological University, Co. Waterford, Waterford City, X91 K0EK, Ireland; Swine Research Group, Agroscope, Posieux, 1725, Switzerland; Eco-Innovation Research Centre, Department of Science, South East Technological University, Co. Waterford, Waterford City, X91 K0EK, Ireland; Teagasc, Animal and Grassland Research and Innovation Centre, Teagasc, Grange, Dunsany, Co. Meath, Ireland; Swine Research Group, Agroscope, Posieux, 1725, Switzerland; Teagasc, Pig Development Department, Animal and Grassland Research and Innovation Centre, Moorepark, Fermoy, Co. Cork, P61 C996, Ireland; Teagasc, Pig Development Department, Animal and Grassland Research and Innovation Centre, Moorepark, Fermoy, Co. Cork, P61 C996, Ireland

**Keywords:** creep feeding, gestation, lactation, microbiome, milk replacer

## Abstract

This study involved 50 sows and was a 2 × 2 factorial arrangement with factors being sow feeding [lactation diet throughout lactation (CON) or gestation diet for the first 5 days of lactation, followed by lactation diet (GEST5)] and creep feeding from day (d) 5 after birth [dry pelleted starter diet (DPS) or liquid mixture of milk replacer and starter diet (LMR+S)]. The study was conducted on two research farms, one in Ireland (IE) and the other in Switzerland (CH). All sows were limit-fed the gestation diet until farrowing. During lactation, both sow treatments followed the same feeding curve with daily digestible energy allocation increasing from 58.1 to 135 MJ between d1 and 28. Sow feed intake, weight and backfat depth and piglet weight and total dry matter disappearance (TDMD) of creep feed during lactation were recorded. On d5 after birth, milk was collected from sows on both farms and sow faeces was collected at CH for short chain fatty acid (SCFA) analysis. Following weaning at d29 ± 0.2 in IE and d25.5 ± 1.3 in CH, pigs were followed until d43 post-weaning (pw) in IE (*n* = 7 pens/treatment) and d14 pw in CH (*n* = 11 pens/treatment). In IE, faecal samples were collected from sows before farrowing and on d5, 12 and 26 after birth and from piglets on d2, 5, 12 and 26 after birth and on d7 and d41 pw for microbiota analysis. Feeding GEST5 did not affect overall sow feed intake, backfat depth or weight loss during lactation on both farms (*P *> 0.05) but reduced the solids, fat and SCFA content of milk and increased faecal SCFA in CH (*P *< 0.05). In CH, LMR+S-fed pigs had higher TDMD than those fed DPS, while in IE, DPS had higher TDMD than LMR+S (*P *< 0.05). However, neither GEST5 nor LMR+S influenced pre- or pw pig growth or diarrhoea prevalence on either farm (*P *> 0.05). In IE, on d5 post-farrowing, GEST5 sows had a higher relative abundance of *Prevotella* and *Succinivibrio* in their faeces compared to CON (*P *< 0.05) and both GEST5 and LMR+S increased bacterial alpha diversity in piglet faeces on d7 pw (*P *< 0.05). In conclusion, while providing a gestation diet during the first 5 days of lactation increased physical feed intake then, it did not increase overall lactation feed intake in sows. Additionally, creep feeding a liquid mixture of milk replacer and starter diet to suckling piglets did not improve pre- or pw pig growth.

## Teaser

Feeding a gestation diet to sows for 5 days post-farrowing did not increase lactation feed intake. Liquid creep feeding did not increase feed intake or growth of suckling piglets but using the automatic delivery system might help in minimising feed wastage when creep feeding.

## Introduction

There is huge potential to increase pre-weaning growth in pigs (Tokach et al. 2020). For example, De Vos et al. (2014) showed that artificially reared piglets can grow 20% faster than sow-reared piglets. Strategies that increase sow milk production and/or increase the intake of supplemental feed by suckling pigs can help to achieve increased growth rates in commercially reared pigs.

Sow milk quality and yield can be improved by optimising sow nutrition (Blavi et al. 2021; Tucker et al. 2021). Increasing lactation feed intake can prevent excessive loss of sow body reserves during lactation which is beneficial for subsequent reproductive performance and sow longevity (Koketsu et al. 1996). The current practice of feeding sows involves providing restricted access to a gestation diet from service to farrowing followed by providing *ad libitum* access to a more energy- and nutrient-dense lactation diet from farrowing until weaning (Theil 2015). Typically, a gestation diet is lower in energy and amino acids but higher in crude fiber than a lactation diet (Tokach et al. 2019). However, the abrupt change from a gestation diet to a lactation diet following farrowing means that it takes some time before the sow acclimatises to eating high quantities of the energy- and nutrient-dense lactation diet (Theil 2015). To facilitate this transition, feeding sows the gestation diet during early lactation could be beneficial. Due to the lower energy and nutrient density of the gestation diet, it is expected that physical feed intake in sows would be increased when the gestation diet is fed in early lactation (Boler et al. 2024; Muller et al. 2025). Our hypothesis was that the bulking effect due to the higher fiber content of the gestation diet would make it necessary for the sows to consume more of this diet to have an equivalent energy intake to that of those on the lactation diet. Promoting higher feed intake early in lactation in this manner (Farmer et al. 1996) should help to increase lactation feed intake thereafter up to weaning (Vignola 2009; Schwennen et al. 2022). The benefits of feeding a high fiber diet to sows around farrowing has previously been demonstrated (Loisel et al. 2013). The insoluble fiber fraction mostly provides bulk in the diet and enhances intestinal peristalsis, thereby reducing constipation (Oliviero et al. 2009; Capuano 2017) and promoting feed intake (Farmer et al. 1996). On the other hand, the soluble fiber fraction increases microbial fermentation and the resultant production of short chain fatty acids (SCFA) in the intestine (Makki et al. 2018). We theorise that these increased amounts of SCFA might be transferred to piglets via sow milk, resulting in improved intestinal health (Lauridsen 2020; Metzler-Zebeli 2021). Furthermore, as most farms have only two diets (gestation and lactation), this strategy of feeding the same gestation diet post-farrowing can be easily applied without the need for a third diet.

Although creep feeding a dry pelleted diet is common on commercial farms, its benefits can be inconsistent. While some studies showed that dry creep feeding increased weaning weight (Pajor et al. 1991; Arnaud et al. 2024) and early post-weaning (pw) feed intake (Muns and Magowan 2018), other studies did not (Sulabo et al. 2010a; Christensen and Huber 2021). The inconsistency in response is thought to be largely due to variation in creep feed intake at both the individual piglet and litter level (Bruininx et al. 2002; Tokach et al. 2020). Offering liquid milk replacer as creep feed can increase feed intake (Christensen and Huber 2021) and weaning weight (Arnaud et al. 2024) in suckling piglets compared to creep feeding a dry pelleted diet. However, providing supplemental milk replacer throughout lactation is not economically viable due to the high cost of milk replacer (Christensen and Huber 2021; Arnaud et al. 2024). Moreover, an important aspect of creep feeding is to expose suckling piglets to plant-based ingredients (commonly found in pw diets), in order to promote intestinal maturity at weaning and facilitate early feed exploration and high feed intake pw (Luo et al. 2021). Therefore, providing a liquid mixture of milk replacer and starter diet to suckling pigs may increase pre-weaning creep feed intake and growth, and provide early exposure to plant-based ingredients prior to weaning. Previous studies manually mixed and fed the liquid milk replacer/liquid creep feed (Byrgesen et al. 2021; Christensen and Huber 2021). However, this approach can be labour-intensive to achieve *ad-libitum* feeding and can lead to uncontrolled fermentation in tanks and troughs, which can lead to loss of nutrients, production of biogenic amines and ethanol, and growth of undesirable bacteria and fungi (Cullen et al. 2021). This can also reduce feed palatability, resulting in lower feed intake and negative impacts on growth and health of pigs (Cullen et al. 2021). In the current study, we hypothesise that creep feeding with a well-maintained automatic liquid delivery system can provide *ad-libitum* access to feed throughout the day, without the negative consequences discussed above.

The hypothesis of the current study was that feeding a gestation diet to sows for 5 days post-farrowing will increase lactation feed intake [both dry matter (DM) and energy] of sows, thereby facilitating increased milk production and hence increased growth and survival of piglets. Furthermore, we hypothesised that offering a liquid mixture of milk replacer and starter diet to suckling piglets will increase their pre-weaning feed intake and growth compared to feeding a dry pelleted starter diet. The effect of gestation diet provision to the sow during early lactation and liquid creep feeding of suckling piglets was expected to be additive in terms of increasing piglet nutrient intake and growth, as well as reducing the excessive loss in sows’ body reserves during lactation. This study was conducted on two research farms using the same diet formulation, milk replacer and automatic liquid feeding system, in order to test the commercial application of the treatments.

## Material and methods

### Study design

This study was conducted between May 2023 and February 2024 at the Teagasc Pig Development Department, Moorepark, Fermoy, Co. Cork, Ireland (IE), and at the Swine Research Group, Agroscope, Posieux, Switzerland (CH). The study was conducted in accordance with the legislation for commercial pig production set out in the European Communities (Welfare of Farmed Animals) regulations 2010 and in Irish legislation (SI no. 311/2010) and with the Swiss Guidelines for Animal Welfare in IE and CH, respectively. In IE, care and use of the animals was approved by the Teagasc Animal Ethics Committee (Approval No. TAEC2022-355) and licensable procedures performed on the animals were authorised by the Irish Health Products Regulatory Authority (project authorisation no. AE19132/P169). In CH, the Swiss Cantonal Committee for Animal Care and Use approved all procedures involving ­animals (approval number: 2023_28_FR).

The experiment involved 50 healthy sows, with two batches studied in IE and two batches studied in CH. The sows were Large White × Landrace (PIC, Hermitage Genetics, Kilkenny, Ireland) in IE and Swiss Large White (Suisag, Sempach, Switzerland) in CH. They had been inseminated with terminal line semen (Topigs Norsvin Tempo, Premier Pig Genetics Limited, Fermoy, Ireland in IE and Swiss Large White, Suisag, Sempach, Switzerland in CH). The sows were transferred on day 109 and day 105 of gestation to individual farrowing crates and loose farrowing pens in IE and CH, respectively.

**Fig. 1.**
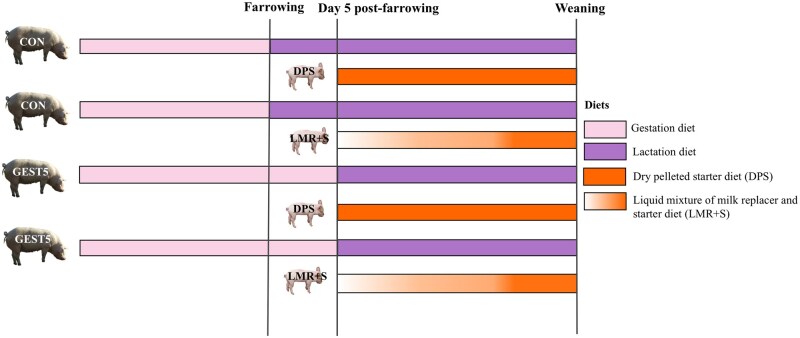
Experimental timeline of the sow and piglet dietary treatments. CON = sows fed with a lactation diet from one day post farrowing until weaning; GEST5 = sows fed with a gestation diet for 5 days post-farrowing followed by a lactation diet until weaning; DPS = suckling piglets provided with dry pelleted starter diet from day 5 to weaning; LMR+S = suckling piglets provided with a liquid mixture of milk replacer and starter diet from day 5 to weaning.

The sows were selected on day 108 (IE) or 110 (CH) of gestation and the distribution of parity of sows was as follows: 26% sows were of parity 0; 38% sows were of parity 1 to 4 and 36% sows were of parity 4 to 7. The sows were blocked within farm by parity (3.4 ± 2.38 in IE and 3.3 ± 1.98 in CH; mean ± SD), number of piglets weaned at the previous lactation (13.5 ± 1.60 in IE and 13.1 ± 1.10 in CH for multiparous sows) and body weight (BW) (286.5 ± 36.2 kg in IE and 282.2 ± 37.3 kg in CH). Each block contained four sows (with similar parity, number of piglets weaned at the previous farrowing and bodyweight). Within block, each sow was randomly assigned to one of four experimental treatments in a 2 × 2 factorial arrangement with factors being sow feeding regime during the first 5 days post-farrowing [gestation diet regime (GEST5) or control regime (CON)] and piglet creep feeding [dry pelleted starter diet (DPS) or liquid mixture of milk replacer and starter diet (LMR+S)]. The CON sows were fed a pelleted lactation diet (Diet 2; Table 1) from one day post-farrowing until service. The GEST5 sows were fed a pelleted gestation diet (Diet 1; Table 1) for 5 days post-farrowing followed by a pelleted lactation diet (Diet 2; Table 1) until service. Both GEST5 and CON sows were provided with the same digestible energy (DE) allowance throughout lactation according to the feed curve detailed below. Both creep feed treatments were provided from day 5 post-farrowing until weaning. Both the sow and creep feed treatments are explained in detail below. Figure 1 shows an experimental timeline of the sow and piglet dietary treatments. On both farms, the litter size within each block was standardized by cross fostering (only within treatment) between 24 and 48 h after farrowing. In IE, piglets’ teeth were clipped within 24 h of birth and on day 5 after birth all piglets were injected with 1 ml of iron (Gleptosil, Ceva Santé Animale, Libourne, France) and the tails were docked. In IE, male piglets remained entire and at weaning in IE (29 ± 0.2 days after birth), a subsample of 296 piglets (8.9 ± 1.33 kg) were followed until day 43 pw. In IE, within each treatment group, single-sex pen groups (9 to 10 male or female pigs of similar weight per pen; *n* = 7 pens/treatment) were formed. In CH, all piglets were injected with 2 ml of iron within 24 h of birth (Feridex 10%, AMAG Pharmaceuticals Inc., Waltham, Massachusetts, USA) but piglets’ teeth were not clipped, and tails were not docked as per routine farm practice. In CH, male piglets were surgically castrated by trained farm staff within two weeks of farrowing using anaesthesia with isoflurane (Piramal Critical Care Inc., Bethlehem, Pennsylvania, USA) administered for ∼90 seconds before the surgical castration was performed and analgesia (Contacera, Zoetis Switzerland GmbH, Zurich, Switzerland) in accordance with Swiss legislation. At weaning in CH (25.5 ± 1.3 days after birth), 281 piglets (7.7 ± 1.40 kg) were followed until day 14 pw. In CH, within each treatment group, mixed-sex pen groups (6 to 7 pigs of similar weight per pen; *n* = 11 pens/treatment) were formed. Details of the housing on the IE and CH farms are included in the supplementary material and methods Section 1.1.

**Table 1.** Composition of the experimental diets (on an air-dry basis; g/kg unless otherwise stated).

Item	Diet specifications				
	Diet 1: Gestation	Diet 2: Lactation	Diet 3: Starter	Diet 4: Link	Diet 5: Weaner
**Ingredient composition (g/kg)**					
** Barley**	747.5	288.6	50.0	77.0	292.7
** Wheat**	0.0	450.0	0.0	100.0	450.0
** Maize**	0.0	0.0	246.9	300.0	0.0
** Soyabean meal**	74.4	173.8	141.1	180.7	150.2
** Full fat soyabean meal**	0.0	0.0	125.0	75.0	50.0
** Soya hulls**	141.9	0.0	0.0	0.0	0.0
** Soya oil**	10.0	49.2	77.5	31.0	23.5
** Whey permeate**	0.0	0.0	200.0	150.0	0.0
** Skim milk powder**	0.0	0.0	125.0	50.0	0.0
** Premix^1^**	1.5	1.5	3.0	3.0	3.0
** L-Lysine HCl**	2.2	5.1	6.4	6.8	6.3
** DL-Methionine**	0.5	1.6	3.6	3.2	2.1
** L-Threonine**	0.9	2.6	3.7	3.3	2.7
** L-Tryptophan**	0.0	0.8	1.4	1.3	0.6
** L-Valine**	0.0	2.6	1.3	1.2	0.7
** Limestone flour**	10.0	11.1	7.0	7.5	10.0
** Mono dicalcium phosphate**	7.2	8.0	5.0	7.0	5.3
** Salt**	4.0	5.0	3.0	3.0	3.0
** Phytase^2^**	0.05	0.05	0.05	0.05	0.05
**Calculated chemical composition (g/kg)^3^**					
** Dry matter**	874.2	879.5	909.7	893.3	876.2
** Crude protein**	130.0	170.0	198.8	190.0	176.0
** Ash**	49.2	48.2	61.6	56.5	44.6
** Crude fat**	27.4	63.5	113.8	61.9	46.5
** Crude fiber**	85.0	29.9	19.6	23.1	31.8
** Neutral detergent fiber**	225.1	125.6	61.3	82.4	129.7
** Acid detergent fiber**	108.5	38.2	24.0	28.6	40.5
** Lysine**	7.8	11.5	16.2	15.0	12.8
** Methionine**	2.4	3.9	7.1	6.2	4.6
** Cystine**	2.5	3.0	2.7	2.9	3.1
** Threonine**	5.4	8.2	10.8	10.0	8.5
** Tryptophan**	1.5	2.8	3.6	3.3	2.6
** Digestible energy (MJ/Kg)**	13.2	15.0	16.2	15.0	14.3
** Net energy (MJ/Kg)**	9.0	10.8	12.1	11.0	10.3
** Standard ileal digestible lysine**	6.6	10.8	15.4	14.2	12.0

1 Premix provided per kilogram of complete diet 1 & 2: Cu from copper sulphate, 15 mg; Fe from ferrous sulphate monohydrate, 70 mg; Mn from manganese oxide, 62 mg; Zn from zinc oxide, 80 mg, I from potassium iodate, 0.6 mg; Se from sodium selenite, 0.2 mg; vitamin A as retinyl acetate, 3.44 mg; vitamin D3 as cholecalciferol, 25 mg; vitamin E as DL-alpha-tocopheryl acetate, 100 mg; vitamin K, 2 mg; vitamin B12, 15 *μ*g; riboflavin, 5 mg; nicotinic acid, 12 mg; pantothenic acid, 10 mg; choline chloride, 500 mg; Biotin, 200 *μ*g; folic acid, 5 mg; vitamin B1, 2 mg; and vitamin B6, 3 mg.

Premix provided per kilogram of complete diet 3, 4 and 5: Cu from copper sulphate, 85 mg; Fe from.

ferrous sulphate monohydrate, 90 mg; Mn from manganese oxide, 47 mg; Zn from zinc oxide, 120 mg; I from potassium iodate, 0.6 mg; Se from sodium selenite, 0.3 mg; vitamin A as retinyl acetate, 2.1 mg; vitamin D3 as cholecalciferol, 25 *μ*g; vitamin E as DL-alpha-tocopheryl acetate, 100 mg; vitamin K, 4 mg; vitamin B12, 15 *μ*g; riboflavin, 2 mg; nicotinic acid, 12 mg; pantothenic acid, 10 mg; choline chloride, 250 mg; vitamin B1, 2 mg; and vitamin B6, 3 mg.

2 Ronozyme HiPhos GT (Inform Nutrition, Whites Cross, Ireland) was included to provide 500 phytase units (FYT) per kg of diet.

3 Calculated chemical composition from book values for ingredients (Sauvant et al. 2004).

### Experimental diets

The gestation, lactation and piglet diets were formulated to meet or exceed recommendations of the National Research Council (NRC 2012). The ingredient composition and nutrient content of the experimental diets are presented in Table 1. In both IE and CH, the gestation diet was fed to sows at a daily feed allowance of 2.65 kg (35 MJ DE) between day 85 of gestation and farrowing. In both IE and CH, feed was provided from electronic sow feeders (Schauer feeding system, Prambachkirchen, Austria) during gestation. After moving the sows to the farrowing accommodation, sows were fed using a computerized feed delivery system (DryExact Pro, Big Dutchman, Vechta, Germany in IE and Schauer Spotmix, Agrotonic GmbH, Prambachkirchen, Austria in CH). The GEST5 sows were fed the gestation diet for 5 days post-farrowing followed by the lactation diet until service, while CON sows were fed with the same lactation diet from one day after farrowing until service. The lactation feeding curve for both GEST5 and CON treatments started at 58 MJ DE/day on day 0 of lactation and gradually increased to 104, 121, 129, and 133 MJ DE/day on days 7, 14, 21, and 26 of lactation, respectively. Although all sows were provided with the same feeding curve, individual sow troughs were inspected twice a day and feed allowance to the individual sow was either increased or decreased based on the level of feed remaining in the trough. This allowed provision of feed to be as close to *ad-libitum* as possible. Any feed refusals were weighed and taken into account when calculating sow feed intake. After weaning, sows on both farms were offered 4 kg of lactation diet per day until service and most were served between 4 and 5 days after weaning.

Dry pelleted starter diet (Diet 3; Table 1) was provided as 3 mm diameter pellets in the DPS creep feeding regime. This diet was also used to prepare LMR+S. The DPS and LMR+S feed troughs on both farms were not accessible to sows. In IE, DPS was provided in a circular feeder trough (Easy pan, Rotecna, Lleida, Spain) which was fixed to the slats at the bottom corner of the heat pad to one side of the sow crate, while the LMR+S feed troughs were installed in the front of the farrowing pen to one side of the sow’s head. In CH, DPS was provided in rectangular metal boxes placed near the piglet nest covered area, while the LMR+S feed troughs were installed to one side of the pens and were protected by a raised wooden box to make the trough inaccessible to the sow. The DPS feeders were checked and refilled manually three times a day to ensure *ad libitum* access but also to minimise feed wastage. The disappearance of DPS for each litter was determined on a weekly basis. In both IE and CH, the LMR+S was ∼15.4% dry matter (DM) and an increasing proportion of starter diet was included in the liquid mixture between day 5 and weaning. The proportion of milk replacer in the LMR+S was 100%, 75%, 50%, 25% and 0% during the periods from day 5 to 9, day 10 to 14, day 15 to 17, day 18 to 21 and day 22 to weaning, respectively. The milk replacer powder (Opticare milk; Swinco B.V, Venray, the Netherlands) contained the following, in descending order of inclusion: sweet whey powder, vegetable oils, porcine dried plasma powder, whey powder, digestible starch, dextrose, hyper-immunised egg powder, soya protein concentrate, hydrolysed wheat gluten and premix of amino acids, vitamins and trace minerals. It contained 11.9 MJ net energy/kg, 21.5% crude protein, 9% fat, 0.1% crude fiber, 6.5% crude ash, 1.8% lysine, 0.46% methionine, 0.7% calcium, 0.55% phosphorus, and 0.7% sodium.

After weaning in IE, pen groups of pigs on each treatment were fed a common series of dry pelleted diets according to their growth stage. Starter diet (Diet 3; Table 1) was provided from the day of weaning to day 7 pw, link diet (Diet 4; Table 1) from day 7 to day 21 pw and weaner diet (Diet 5; Table 1) from day 21 to day 43 pw. After weaning in CH, pigs were fed the starter diet (Diet 3) from the day of weaning to day 14 pw. Representative feed samples from both farms were analysed for DM, ash, crude protein, crude fat, crude fiber, neutral detergent fiber and acid detergent fiber as previously described by Palumbo et al. (2023).

### Pre-weaning liquid feeding system

An automated liquid feed delivery system (Babyfeed; Schauer Agrotronic GmbH) was used to provide the LMR+S to suckling piglets on both farms. Two fresh mixes of LMR+S were prepared daily at 0835 and 1645 h by mixing the milk replacer powder and starter diet with warm water at 55 °C. Ten feeding cycles were programmed between 0930 and 0400 h in IE and between 0930 and 0000 h in CH. During each cycle, the in-situ trough sensors checked the level of liquid feed present in the troughs five times. Whenever the liquid feed was below the sensor level, fresh LMR+S was delivered to the trough and the amount delivered was recorded by the system. Therefore, there was potential for each trough to be supplied with fresh LMR+S ∼50 times in a 24 h period. Weekly cleaning of the system was as described by Vasa et al. (2024a) and is described in detail in the supplementary material and methods ­Section 1.2.

### Data recording, sampling & laboratory analysis

#### Sow feed intake, body weight and backfat depth

Feed delivered to each sow was recorded on a daily basis and average daily feed intake (ADFI) from day 0 to 5 post-farrowing, day 6 to 14 post-farrowing and day 15 to weaning were calculated. Using these data and the chemical composition of the diets fed, average daily DE intake, average daily net energy intake, average daily standard ileal digestible lysine intake, average daily fat intake, average daily protein intake and average daily crude fiber intake were calculated for the same periods as above.

Sow BW and backfat depth were recorded at day 109 of gestation, weaning and service (day 5 pw). Sow BW was recorded using an electronic weighing scale (EziWeigh 7i, O’Donovan Engineering, Coachford, Ireland in IE and Rhewa 82 basic, Grüter Waagen GmbH, Eschenbach, Switzerland in CH). The empty farrowing weight of sows was calculated as described in NRC (1998) and backfat depth was determined using a Renco Lean-Meater (Renco Corporation, Minnesota, USA) as described by Arnaud et al. (2024) and as explained in detail in the supplementary material and methods Section 1.3.

#### Sow milk sampling and analysis

Sow milk samples were collected from all sows in IE and CH on day 5 post-farrowing. After separating the piglets from the sow for 1.5 h, a 2 ml intramuscular injection of oxytocin (AgriHealth, Monaghan, Ireland in IE and MSD Animal Health GmbH, Luzern, Switzerland in CH) was administered to induce milk let-down and the area surrounding the teats was cleaned using alcohol-infused wipes (ClearKlens IPA Swipe, Diversey Europe Operations BV, Utrecht, The Netherlands) prior to collecting milk samples. Milk samples were collected from all of the teats until the end of milk letdown and were stored at −80°C for compositional and microbiome analysis. For compositional analysis, milk samples were defrosted at room temperature. Once fully thawed, samples were mixed by inverting several times. Each sample was analysed for total solids, lactose, fat and protein content by near-infrared absorption (Dairyspec FT, Bentley Instruments Inc., Chaska, Minnesota, USA). The sow milk SCFA profile was analysed using gas-liquid chromatography (Gaschromatograph Series Agilent 6890, Agilent Technologies 2000, Santa Clara, California, USA) as described by Palumbo et al. (2023). Microbiota analysis of the sow milk is described below.

#### Sow faecal scoring and DM content

To monitor the prevalence of constipation, sow faecal consistency was visually scored each day by one evaluator during the first 5 days post-farrowing in both IE and CH. A 6-grade scoring system (Oliviero et al. 2009) was used as follows: 0 (absence of faeces), 1 (dry and pellet-shaped), 2 (between dry and normal), 3 (normal and soft, but firm and well formed), 4 (between normal and wet, still formed but not firm), 5 (very wet faeces, unformed and liquid). A sow faecal score of 0–2 was considered indicative of constipation and the average percentage of sows experiencing constipation during the first 5 days post-farrowing was calculated. In addition to the visual scoring, sow faecal samples (∼200 g) were collected in CH on day 1, 3 and 5 post-farrowing and oven dried for 21 h at 63 °C and then for 3 h at 106 °C. The faecal samples were weighed before and after oven drying to determine the DM content and the average faecal DM for all three days post-farrowing was calculated.

#### Mortality, medication usage and diarrhoea prevalence

All piglet deaths, antibiotic and anti-inflammatory usage (ml/litter/pen) and number of clinical cases of disease per litter (number of pigs that were treated on one or more occasion) were recorded. In IE, the only antibiotic used was Unicillin (Procaine Benzlypenicllin, 300 mg/ml injection, Univet, Cootehill, Cavan, Ireland) and the only anti-inflammatory used was Loxicom (5 mg/ml injection, Norbrook, Monaghan, Ireland). In CH, Betamox (Norbrook) was used as the antibiotic and Rifen (Streuli Tiergesundheit AG, Uznach, Switzerland) was used as the anti-inflammatory. The percentage mortality between 48 h after birth and weaning for each litter was calculated as the percentage of deaths based on the litter size per sow after cross-fostering. Visual scoring of faecal consistency at pen level was performed by one evaluator as described by Casey et al. (2007) on day 12 and 20 after birth, at weaning and on day 2, 4, 6, 8, 10 and 12 pw. A full description of the scoring system is provided in the supplementary material and methods Section 1.4. Diarrhoea prevalence was calculated for the pre-weaning period (day 12 to weaning) and the early pw period (day 2 to 12 pw).

#### Pre-weaning growth performance

In IE, suckling piglets were weighed individually at birth and on day 5, 12, 20 and 28 after birth using an electronic weighing scale (Defender 3000 XtremeW, O’Donovan Engineering). In CH, suckling piglets were weighed individually at birth and on day 5, 12, 19 and 25 after birth using an electronic weighing scale (ICS429, Mettler-Toledo GmbH, Greifensee, Switzerland). In both IE and CH, creep feed disappearance (feed offered minus feed refusals) for DPS was recorded between each pig weighing. In both IE and CH, creep feed disappearance of LMR+S was recorded daily by the liquid feeding system. The total DM disappearance (TDMD) during the entire pre-weaning period and the average daily DM disappearance between each pig weighing were calculated and expressed on a per pig per litter basis. Average daily gain (ADG) of piglets was calculated using the average individual piglet BW of each litter on each weighing day. The coefficient of variation (CV) of within litter piglet BW was calculated by dividing the standard deviation of piglet BW within each litter by the mean piglet BW of the litter and multiplying this by 100.

#### Post-weaning growth performance

In IE, pen groups were weighed on day 0 (weaning), 7, 14, 21, and 43 pw using an electronic weighing scales (EziWeigh 7i, O’Donovan Engineering). In CH, pen groups were weighed on day 0 (weaning), 7, and 14 pw using an electronic weighing scales (82 basic T5646 TC 7033, Grüter Waagen GmbH, Eschenbach, Switzerland). Feed disappearance in each pen was recorded for each interval between the weighing days. Using these data, the ADFI, ADG and gain to feed ratio of each pen group was calculated.

#### Faecal sampling and analysis

Faecal samples from the first batch of sows and piglets in IE (3 sows per treatment) were collected for microbiota analysis. Faecal samples from sows were collected on day 109 of gestation and on day 5, 12 and 26 after farrowing. Fifteen focal piglets per treatment (5 from each sow per treatment, median birth weight for their litter and balanced for sex) were chosen for faecal sampling. Sterile viscose swabs (swab in tube 101 x 16.5 mm, Sarstedt, Co Wexford, Ireland) were used to collect rectal swabs from piglets on day 2, 5, 12 and 26 after birth. Faecal samples were collected from the same 15 focal pigs/treatment on day 7 and 41 pw. Whenever possible, freshly voided faecal samples were collected from the pig and if this was not possible, samples were obtained by digital rectal stimulation, with gloves changed between each sampled pig. All faecal samples were carefully placed into sterile 50 ml plastic tubes and put on ice. Sub-samples were then transferred into sterile 2 ml tubes, snap frozen in liquid nitrogen (within 5 minutes of collection) and stored at -80°C until DNA extraction. Only rectal swabs/faecal samples from ∼10 focal pigs per treatment (median birth weight for their litter and balanced for sex) which were observed to be consuming DPS or LMR+S creep feed and remained healthy until day 41 pw were subsequently selected for microbiota analysis. Live observations (3-minute instantaneous scan sampling) as described by Vasa et al. (2024b) were used to determine the individual piglets which were observed to consume DPS or LMR+S creep feed. Live observations were conducted on day 13, 20 and 26 after birth and piglets which were observed to consume DPS or LMR+S creep feed at least once on each observation day were selected for microbiota analysis.

Sow faecal samples were collected in CH on day 5 post-farrowing and stored at -20°C for SCFA analysis. The SCFA profile in the sow faeces was determined by high-performance liquid chromatography as previously described by Girard et al. (2021) and as described in the supplementary material and methods Section 1.5.

#### DNA extraction and sequencing

The DNA extraction and sequencing was as previously described by Rattigan et al. (2023) and Cullen et al. (2022). Total bacterial DNA was extracted from rectal swabs and faecal samples using the Qiagen QIAamp Fast DNA Stool Mini Kit (Qiagen, Hilden, Germany). The manufacturer’s instructions were followed, except that a bead beating step was added and the lysis temperature was increased to 95 °C, to increase DNA yield. The volume of buffer ATE (elution buffer) was also reduced from 200 to 50 µl and it was allowed to sit on the filter membrane of the spin column for 5 min at room temperature prior to centrifugation (both to maximise DNA concentration). For faecal samples, 0.25 g of faecal material was used and for the rectal swabs, the swab tip was broken off and used for DNA extraction. Total bacterial DNA from sow milk samples collected from the first batch of sows (3 sows per treatment) was extracted using the Qiagen DNeasy PowerFood Microbial Kit (Qiagen) according to the manufacturer’s instructions with some modifications as follows. The initial homogenisation step was omitted, and 1.8 ml of sow milk was added to the 2 ml collection tube in triplicate in order to process 5.4 ml sow milk. The duration of the first centrifugation step was increased to 15 min to maximise pellet formation and a bead beating step was added. The volume of the elution buffer was also reduced from 100 to 20 µl and it was allowed to sit on the filter membrane of the spin column for 5 min at room temperature prior to centrifugation (both to maximise DNA concentration).

Library preparation and sequencing were performed by Macrogen Inc. (Seoul, South Korea) after an initial DNA concentration check with the Invitrogen Qubit 4 Fluorometer (Thermo Fisher Scientific, Waltham, Massachusetts, USA) using a Qubit dsDNA Quantification Assay Kit (Thermo Fisher Scientific). The 16S libraries were prepared according to the Illumina 16S Metagenomic Sequencing Library protocols to amplify the V3-V4 region of the 16S rRNA gene. The initial amplicon PCR mixture contained 5 ng of DNA, 1x reaction buffer, 1 mM dNTPs, 500 nM of each of the forward and reverse primers, and Herculase II fusion DNA polymerase (Agilent Technologies, Santa Clara, California, USA). The conditions for the initial PCR were 3 min at 95 °C, followed by 25 cycles of 30 sec at 95 °C, 30 sec at 55 °C and 30 sec at 72 °C, followed by a 5 min final extension at 72 °C. The primer pair with Illumina adapter overhang sequences used for the initial PCR were as follows: V3-F: 5’-TCGTCGGCAGCGTCAGATGTGTATAAGAGACAGCCTACGGGNGGCWGCAG-3’, V4-R: 5’-TCTCGTGGGCTCGGAGATGTTATAAGAGACAGGACTACHVGGGTATCTAATCC-3’. The initial PCR products were purified with AMPure beads (Agencourt Bioscience, Beverly, Massachusetts, United States). Following purification, an index PCR was performed with 2 µL of the initial PCR products for final library construction containing Nextera XT indices. The same PCR conditions were used as the initial PCR, except that the number of cycles was reduced to 10. The indexed PCR products were purified with AMPure beads and quantified using qPCR according to the qPCR Quantification Protocol Guide (KAPA Library Quantification kits for Illumina Sequencing platforms) and library sizes were measured using the TapeStation D1000 ScreenTape (Agilent Technologies, Waldbronn, Germany). Paired-end (2 × 300 bp) sequencing was performed using the MiSeq platform (Illumina, San Diego, California, USA). The 16S rRNA gene sequences were processed in R (version 4.0.2) using DADA2 (version 1.20.0). Quality checks of both forward and reverse reads were carried out using FASTQC and optimal filtering and trimming parameters were identified using FIGARO v3.0. Primer removal and quality filtering and trimming of read pairs was carried out using the filterandTrim function in DADA2. Following de-replication and read pair merging an amplicon sequence variant (ASV) table was constructed. Chimeric sequences from de-noised data were subsequently removed. Taxonomic classification of ASVs was carried out using the SILVA taxonomic database (v.139.1). Sample metadata, sequence taxonomy, and ASVs were combined into a phyloseq object (version 1.34.0). Potential contaminants were identified and removed using decontam v.1.12.0. Species/strain/serotype level identification of relevant genera was performed by searching the sequence in the Basic Local Alignment Search Tool nucleotide database of the U.S. National Center for Biotechnology Information (available at: https://blast.ncbi.nlm.nih.gov/Blast.cgi).

### Statistical analysis of data

Statistical analyses of data (except microbiota data) were performed using SAS version 9.4 (SAS Institute Inc. Cary, North Carolina, USA). All data were tested for normality by inspecting the normal distribution and residuals plots using the univariate procedure. Initially, data from both the IE and CH farms were combined and analysed, with two scenarios: the farm included as a fixed effect or as a random effect. However, the data obtained from each farm were very different and some results were contradictory between farms, as explained in the discussion section. For this reason and to discuss differences between farms, it was decided (in consultation with a statistician) to analyse and present the data from each farm separately. Pre-weaning and pw growth performance indicators which were recorded at multiple time points were analysed as repeated measures using the PROCMIXED procedure for a 2 × 2 factorial arrangement. The model included sow feeding regime (CON, GEST5), creep feeding regime (DPS, LMR+S) and their associated interactions as fixed effects with day as the repeated variable. The ‘slice’ option was used to obtain the effects at each day. Block was included as a random effect and piglet birth weight (for pre-weaning parameters) and weaning weight (for pw parameters) were included as co-variates, when significant in the model. The litter/sow was the experimental unit prior to weaning and pen group was the experimental unit pw. Sow BW and backfat depth parameters were analysed using the same procedure as described above, with litter size after cross-fostering included as a co-variate in the model, when significant. The sow feed intake parameters were also analysed using repeated models as described above with sow feeding regime (CON or GEST5) and day as fixed effects. Sow milk composition, faecal SCFA profile and faecal DM content were analysed with sow feeding regime as the fixed effect. In all models above, the appropriate co-variance structure as indicated by the model fit statistics was applied and Tukey-Kramer adjustment was used to account for multiple comparison of means. The percentage of sows experiencing constipation, and prevalence of diarrhoea in piglets during the lactation period and the early pw period were not normally distributed and were analysed using PROC GLIMMIX using a binomial distribution. Pre-weaning mortality percentage per litter was analysed using PROC GLIMMIX using a multinomial distribution.

For microbiota data, alpha (observed, Shannon and Inverse-Simpson indices) and beta (Bray-Curtis distance) diversity metrics were estimated using the microeco and phyloseq packages in R (version 4.3). Comparisons of various diversity metrics between treatment groups was carried out using the Adonis function in the Vegan R package, Wilcox rank sum tests in the microeco R package and lm function from the stats library. Differential abundance estimates between treatment groups (only interaction effect for piglet faecal samples) was carried out using both the LinDa and DeSeq2 R packages. Visualisation of results was performed using the R ggplot2 package. Significant differences were considered when *P *< 0.05, with 0.05 < *P *≤ 0.1 considered as tendencies.

## Results

The analysed chemical composition of the experimental diets used in IE and CH are presented in Supplementary Tables S1 and S2, respectively and the calculated chemical composition of the experimental diets is presented in Table 1. The differences between the analysed chemical composition values of diets used on both farms were minimal.

### Sow feed intake, BW and backfat depth

The effect of sow feeding treatment on ADFI of sows on both research farms is presented in Table 2 and on daily DE, net energy, crude protein, standard ileal digestible lysine, crude fat and crude fiber intakes in Supplementary Table S3. In IE from day 0–5 post-farrowing, GEST5 sows tended to have higher ADFI (*P *= 0.06), higher average daily crude fiber intake (*P *< 0.05) and lower average daily crude protein, standard ileal digestible lysine and fat intake (*P *< 0.05) than CON sows. In CH, during the same period, GEST5 sows had higher ADFI and average daily crude fiber intake (*P *< 0.05) but lower average daily fat intake (*P *< 0.05) and tended to have lower average daily standard ileal digestible lysine intake (*P *= 0.08) compared to CON sows. However, during the same period, the average daily DE intake and the average daily net energy intake did not differ between GEST5 and CON sows on both farms (*P *> 0.05). There was no residual effect of sow feeding treatment on ADFI, average daily DE, net energy, crude protein, standard ileal digestible lysine, crude fiber and fat intake from day 6 to 14 post-farrowing or from day 15 post-farrowing to weaning on both farms (*P *> 0.05).

**Table 2.** Effect of sow feeding treatment (CON or GEST5) on sow feed intake from farrowing to weaning on the Irish (IE) and Swiss (CH) research farms [least square means ± pooled standard errors of the mean (SEM)].

IE				
Sow feeding	CON	GEST5	SEM	*P* value
**Number of sows/litters**	13	14		
**Average daily feed intake (kg/sow/day)**				
** Day 0–5**	5.3	6.0	0.24	0.06
** Day 6–14**	7.8	7.4	0.25	0.36
** Day 15-weaning^1^**	8.8	8.4	0.21	0.31
** Overall**	7.0	7.1	0.20	0.82

CH				
Sow feeding	CON	GEST5	SEM	*P* value
**Number of sows/litters**	11	12		
**Average daily feed intake (kg/day/sow)**				
** Day 0–5**	4.6	5.4	0.22	0.03
** Day 6–14**	7.2	7.1	0.16	0.69
** Day 15-weaning^1^**	7.1	7.5	0.33	0.46
** Overall**	6.3	6.6	0.19	0.26

Abbreviations: CON = sows fed with a lactation diet from one day post-farrowing until weaning; GEST5 = sows fed with a gestation diet for 5 days post-farrowing followed by a lactation diet until weaning; IE = research farm in Ireland; CH = research farm in Switzerland.

1 Weaning in IE = 29.0 ± 0.1 day of lactation; Weaning in CH = 25.5 ± 1.3 day of lactation.

There was no sow feeding × creep feeding interaction effect or associated main effect on sow BW, backfat depth and change in sow BW or backfat depth at any time period on both farms (Supplementary Table S4; *P *> 0.05).

### Sow milk composition and SCFA profile

The effect of sow feeding treatment on sow milk composition and SCFA profile on day 5 post-farrowing on both research farms is presented in Table 3. There was no effect of sow feeding treatment on milk solids, lactose, protein or fat % on the IE farm (*P *> 0.05). Only formate, acetate and butyrate were found at detectable levels in the sow milk samples. In IE, CON sows’ milk had a higher butyrate concentration than that of GEST5 sows (*P *< 0.05), but the concentration of total SCFA, formate and acetate did not differ between treatments (*P *> 0.05). In CH, GEST5 sows’ milk had lower solids and fat % (*P *< 0.05) and tended to have lower protein % (*P *= 0.09) than CON sows, but the lactose % was not affected by treatment (*P *> 0.05). In CH, CON sows’ milk had a higher concentration of total SCFA, formate and butyrate (*P *< 0.05) and tended to have a higher acetate concentration (*P *= 0.05) than that of GEST5 sows.

**Table 3.** Effect of sow feeding treatment (CON or GEST5) on sow milk composition and short chain fatty acid profile on day 5 post-farrowing on the Irish (IE) and Swiss (CH) research farms [least square means ± pooled standard errors of the mean (SEM)].

IE				
Sow feeding	CON	GEST5	SEM	*P* value
**Number of sows**	13	14		
**Solids (%)**	18.5	18.1	0.46	0.57
**Lactose (%)**	4.9	4.9	0.16	0.95
**Protein (%)**	4.6	4.8	0.10	0.32
**Fat (%)**	7.3	6.7	0.50	0.38
**Total short chain fatty acids (µmol/g)**	2.2	2.1	0.12	0.54
** Formate (*µ*mol/g)**	0.23	0.22	0.01	0.61
** Acetate (*µ*mol/g)**	1.83	1.79	0.11	0.75
** Butyrate (*µ*mol/g)**	0.10	0.05	0.01	0.04

CH				
Sow feeding	CON	GEST5	SEM	*P* value
**Number of sows**	11	12		
**Solids (%)**	19.4	17.8	0.33	<0.01
**Lactose (%)**	4.5	4.6	0.09	0.27
**Protein (%)**	5.4	5.0	0.15	0.09
**Fat (%)**	7.8	6.6	0.31	0.01
**Total short chain fatty acids (*µ*mol/g)**	3.03	2.44	0.16	0.01
** Formate (*µ*mol/g)**	0.27	0.23	0.01	0.02
** Acetate (*µ*mol/g)**	2.48	2.11	0.13	0.05
** Butyrate (*µ*mol/g)**	0.23	0.07	0.02	<0.01

Abbreviations: CON = sows fed with a lactation diet from one day post-farrowing until weaning; GEST5 = sows fed with a gestation diet for 5 days post-farrowing followed by a lactation diet until weaning; IE = research farm in Ireland; CH = research farm in Switzerland.

### Sow faecal visual scoring and DM content

In IE, there was no effect of sow feeding treatment on the percentage of sows experiencing constipation during the first 5 days post-farrowing, with 28.6% of GEST5 and 29.2% of CON sows experiencing constipation (SEM = 5.52; *P *= 0.93).

However, in CH, there was an effect of sow feeding treatment, with 26.7% of GEST5 and 56.7% of CON sows experiencing constipation during the first 5 days post-farrowing (SEM = 6.20; *P *< 0.05). However, no effect of sow treatment on sow faecal DM was found in CH during this period, with the faeces of GEST5 sows having 29.0% DM and CON having 30.5% DM (SEM = 1.12; *P *= 0.36).

### Piglet mortality, medication usage and prevalence of diarrhoea

There was no sow feeding × creep feeding interaction effect or any associated main effect on the percentage of piglet mortality between 48 h after birth and weaning in either IE or CH (*P *> 0.05). The average mortality during this period was 13.5 ± 4.5% and 3.9 ± 1.7% in IE and CH, respectively. A full description of the deaths/removals in both IE and CH per treatment is included in the Supplementary results Section 2.1. There was no sow feeding × creep feeding interaction effect or any associated main effect on antibiotic or anti-inflammatory usage or number of clinical cases of disease per litter in suckling piglets (Supplementary Table S5) or on prevalence of diarrhoea in suckling (Supplementary Table S5) or weaned piglets (Supplementary Table S6) in both IE and CH (*P *> 0.05).

### Pre-weaning piglet feed intake and growth performance

The sow feeding × creep feeding interaction and their associated main effects on feed intake and growth performance of suckling piglets for both research farms are presented in Table 4. In IE, there was no sow feeding × creep feeding interaction effect or any associated main effect on BW of suckling piglets from day 0 to weaning (*P *> 0.05). In IE, while there was no sow feeding × creep feeding interaction or main effect of sow feeding, there was an effect of creep feeding on TDMD during the entire pre-weaning period. Piglets fed with DPS had higher TDMD than LMR+S-fed piglets (573 vs 286 g/pig; SEM = 41.8; *P *< 0.05). In IE, there was no sow feeding × creep feeding interaction effect or any associated main effect on ADG of suckling piglets at any time period from day 0 to weaning (*P *> 0.05). However, piglets supplemented with LMR+S tended to have higher ADG from day 5 to 12 than piglets supplemented with DPS (254 vs 217 g/day; SEM = 13.5; *P *= 0.07).

**Table 4.** Effect of sow feeding treatment (CON or GEST5), creep feeding treatment (DPS or LMR+S) and their associated interactions on feed intake and growth performance of suckling piglets on the Irish (IE) and Swiss (CH) research farms [least square means ± pooled standard errors of the mean (SEM)].

IE								
Sow feeding	CON		GEST5		SEM	*P-*value		
Creep feeding	DPS	LMR+S	DPS	LMR+S		*Sow feed*	*Creep feed*	*Sow feed × Creep feed*
**Number of sows/litters**	7	6	7	7				
**Litter size at birth**	15.4	15.0	15.7	15.1				
**Litter size at weaning**	13.6	13.0	13.1	13.3				
**Bodyweight (kg)**								
** Day 0 (birth)**	1.44	1.42	1.38	1.45	0.06	0.82	0.70	0.80
** Day 5**	2.39	2.32	2.23	2.26	0.13	0.42	0.88	0.83
** Day 12**	3.49	3.68	3.38	3.72	0.31	0.92	0.41	0.85
** Day 20**	6.27	6.78	5.89	6.26	0.29	0.14	0.14	0.24
** Day 28 (weaning^1^)**	8.50	8.92	8.07	8.24	0.35	0.12	0.40	0.38
** Overall**	4.42	4.62	4.19	4.39	0.20	0.25	0.32	0.99
**TDMD (DM basis, g/pig)**	630	322	515	249	59.3	0.13	<0.01	0.74
**ADG (g/pig/day)**								
** Day 0–5**	156	149	143	132	14.5	0.32	0.52	0.68
** Day 5–12**	223	258	212	249	19.1	0.60	0.07	0.31
** Day 12–20**	275	310	260	271	18.4	0.16	0.22	0.31
** Day 20–28**	278	267	275	247	18.9	0.54	0.31	0.63
** Overall**	233	246	222	225	12.9	0.23	0.56	0.69

CH								
Sow feeding	CON		GEST5		SEM	*P*-value		
Creep feeding	DPS	LMR+S	DPS	LMR+S		*Sow feed*	*Creep feed*	*Sow feed × Creep feed*
**Number of sows/litters**	6	5	6	6				
**Litter size at birth**	12.7	13.6	12.7	13.2				
**Litter size at weaning**	11.8	12.2	12.3	12.8				
**Bodyweight (kg)**								
** Day 0 (birth)**	1.46	1.37	1.51	1.39	0.10	0.64	0.29	0.71
** Day 5**	1.99	2.20	2.35	2.00	0.14	0.52	0.56	0.24
** Day 12**	3.91	3.97	4.04	3.84	0.23	0.95	0.72	0.94
** Day 19**	5.68	6.30	6.32	5.49	0.28	0.79	0.69	0.11
** Day 25 (weaning^1^)**	7.59	8.12	7.94	6.97	0.36	0.28	0.52	0.15
** Overall**	4.13	4.37	4.43	3.94	0.17	0.72	0.99	0.62
**TDMD (DM basis, g/pig)**	51	131	30	116	24.4	0.47	<0.01	0.89
**ADG (g/pig/day)**								
** Day 0–5**	128	157	165	157	14.2	0.20	0.50	0.28
** Day 5–12**	275	252	242	264	22.0	0.65	0.95	0.73
** Day 12–19**	253	332	324	236	41.5	0.77	0.90	0.27
** Day 19–25**	273	259	232	211	25.3	0.10	0.49	0.33
** Overall**	232	250	241	217	12.2	0.33	0.77	0.10

Abbreviations: CON = sows fed with a lactation diet from one day post farrowing until weaning; GEST5 = sows fed with a gestation diet for 5 days post-farrowing followed by a lactation diet until weaning; DPS = suckling piglets provided with dry pelleted starter diet from day 5 to weaning; LMR+S = suckling piglets provided with a liquid mixture of milk replacer and starter diet from day 5 to weaning; IE = research farm in Ireland; CH = research farm in Switzerland; TDMD = Total dry matter disappearance during the entire pre-weaning period: DM = dry matter; ADG = average daily gain.

1 Weaning in IE = 29.0 ± 0.1 day of lactation; Weaning in CH = 25.5 ± 1.3 day of lactation.

In CH, there was no sow feeding × creep feeding interaction effect or any associated main effect on BW, TDMD or ADG of suckling piglets from day 0 to weaning (*P *> 0.05), except for a creep feeding main effect on TDMD. Piglets creep-fed with LMR+S had higher TDMD than those fed DPS (123 vs 41 g/pig; SEM = 17.3; *P *< 0.05). The effect of sow feeding × creep feeding interaction and their associated main effects on average daily DM disappearance and within litter CV of BW for suckling piglets on both research farms are presented in Supplementary Table S5 with treatment differences described in the Supplementary results Section 2.2. In general, treatment differences for average daily DM disappearance were similar to those for TDMD.

### Post-weaning piglet feed intake and growth performance

There was no sow feeding × creep feeding interaction effect or any associated main effect on BW, ADFI, ADG or gain to feed ratio of weaned pigs from weaning to day 43 pw in IE or from weaning to day 14 pw in CH (Supplementary Table S6; *P *> 0.05).

### Sow faecal and milk microbiota diversity and composition

**Fig. 2.**
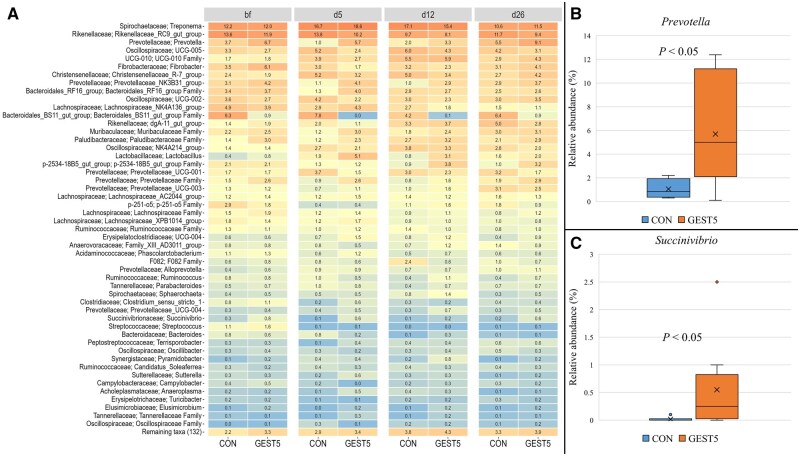
Effect of sow feeding treatment (CON or GEST5) on relative abundance (%) of bacteria genera in the sow faecal samples collected on day 109 of gestation (before farrowing; bf) and on day (d) 5, 12 and 26 after farrowing on the Irish research farm, where CON = sows fed with a lactation diet from one day post-farrowing until weaning; GEST5 = sows fed with a gestation diet for 5 days post-farrowing followed by a lactation diet until weaning. a) Heatmap of the mean relative abundance (%) of the 50 most abundant genera per treatment in the sow faeces. b) Effect of treatment on mean relative abundance (%) of the *Prevotella* genus on day 5 after farrowing. c) Effect of treatment on mean relative abundance (%) of the *Succinivibrio* genus on day 5 after farrowing. Significant differences between treatments are indicated as *P* < 0.05.

There was no effect of sow feeding treatment on any of the faecal alpha diversity indices at any sampling time point (Supplementary Fig. S1a). The effect of sow feeding treatment on beta diversity (Bray-Curtis distance) of the sow faecal samples at the four sampling time points is presented in Supplementary Fig. S1b. Faecal microbiota beta diversity for CON and GEST5 sows differed at day 5 after birth (*P *< 0.05) but at no other sampling point (*P *> 0.05). A total of 14 phyla, 78 families and 196 genera were identified in sow faecal samples across all sampling time points, with *Bacteroidota* and *Firmicutes* being the predominant phyla. The mean relative abundances of the 50 most abundant genera at each sampling time point by sow treatment group are presented in Fig. 2a. There were no sow feeding treatment differences in faecal relative abundance for any genera on day 109 of gestation (*P *> 0.05). On day 5 after birth, the relative abundances of *Prevotella* (Fig. 2b), *Succinivibrio* (Fig. 2c) and *Megasphaera* (data not shown due to relative abundance < 1%) were higher in the faeces of GEST5 than CON sows (*P *< 0.05). On day 12 after birth, the relative abundance of an unidentified genus from the family p-2534-18B5_gut_group was higher in the faeces of GEST5 than CON sows (3.8 vs 0.7 ± 0.45%; *P *< 0.05). There was no effect of sow feeding treatment on faecal relative abundance of any genera on day 26 after birth (*P *> 0.05).

There was no effect of sow feeding treatment on any of the bacterial alpha diversity indices or on beta diversity (Bray-Curtis distance) in the sow’s milk on day 5 after birth (*P *> 0.05). A total of 9 phyla and 111 genera were identified in the sow milk samples, with *Firmicutes* and *Proteobacteria* being the predominant phyla. There were no treatment differences in relative abundance for any of the genera found in the milk samples (*P *> 0.05).

### Piglet faecal microbiota diversity and composition

The effects of sow feeding and creep feeding on alpha diversity of the piglet faecal microbiota are presented in Supplementary Fig. S2. There was no sow feeding × creep feeding interaction effect at any pre-weaning time point for the piglet rectal samples (*P *> 0.05). At day 2 after birth, there were no differences in the faecal alpha diversity indices between piglets from GEST5 and CON sows (*P *> 0.05). At day 5 after birth, piglets from GEST5 sows tended to have higher observed faecal alpha diversity than piglets from CON sows (*P *= 0.08). At day 12 after birth, there was no sow feeding treatment main effect but there was a main effect of creep feeding, whereby LMR+S-fed piglets had higher observed faecal alpha diversity than DPS-fed piglets (*P *< 0.05). There were no main effects of sow feeding or creep feeding treatment on faecal alpha diversity indices on day 26 after birth (*P *> 0.05). On day 7 pw, there was a sow feeding × creep feeding interaction, whereby feeding LMR+S, but not DPS, to piglets originating from CON, but not GEST5, sows increased all three faecal alpha diversity indices (*P *< 0.05). On day 7 pw, there was a main effect of creep feeding treatment, where LMR+S-fed piglets had higher faecal alpha diversity (all three indices) than DPS-fed piglets (*P *< 0.05). On day 7 pw, there was a main effect of sow feeding treatment, where piglets originating from GEST5 sows had a higher Shannon index than piglets originating from CON sows (*P *< 0.05). On day 41 pw, there was no treatment effect on piglet faecal alpha diversity (*P *> 0.05).

**Fig. 3.**
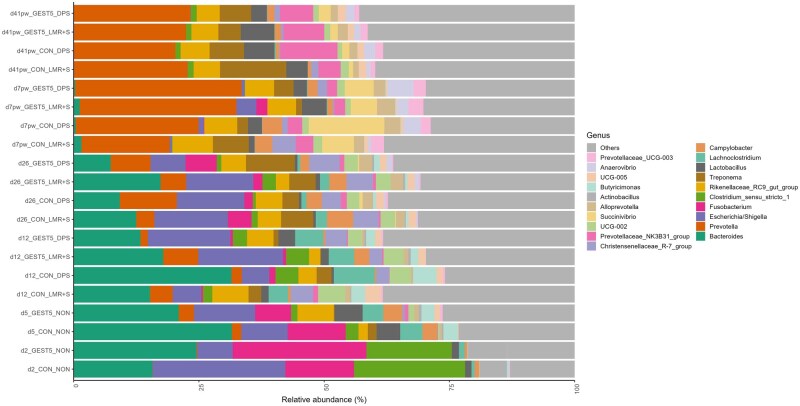
Effect of sow feeding treatment (CON or GEST5) and creep feeding treatment (DPS or LMR+S) on the mean relative abundances (%) of the 20 most abundant genera per treatment group in the piglet faecal samples collected on day (d) 2, 5, 12 and 26 after birth and day 7 (d7pw) and 41 post-weaning (d41pw) on the Irish research farm. CON = sows fed with a lactation diet from one day post farrowing until weaning; GEST5 = sows fed with a gestation diet for 5 days post-farrowing followed by a lactation diet until weaning; DPS = suckling piglets provided with dry pelleted starter diet from day 5 to weaning; LMR+S = suckling piglets provided with a liquid mixture of milk replacer and starter diet from day 5 to weaning; NON = no creep feeding treatment.

The effect of treatment combinations on piglet faecal beta diversity at each sampling time point is represented in Supplementary Fig. S3. In general, the faecal samples clustered by age of the pigs. At days 2 and 5 after birth, the beta diversity of samples from piglets from GEST5 sows differed from those from piglets from CON sows (*P *< 0.05). At day 12 after birth, there was a sow feeding × creep feeding interaction, where the beta diversity of faecal samples from LMR+S-fed piglets from CON sows differed from those of DPS-fed piglets from CON sows (*P *< 0.05). At day 26 after birth, there was no difference in beta-diversity between any treatment groups (*P *> 0.05). At day 7 pw, the beta diversity of LMR+S-fed piglets originating from CON sows differed from that of DPS-fed piglets originating from GEST5 sows (*P *< 0.05). At day 41 pw, there was no difference in beta-diversity between any treatment groups (*P *> 0.05). A total of 15 phyla, 104 families and 309 genera were identified in piglet faecal samples across all time points. On day 2 after birth, *Firmicutes* and *Proteobacteria* were the predominant phyla and *Bacteroidota* and *Firmicutes* predominated at all other time points. The mean relative abundance of the 20 most abundant genera by treatment group at each time point is presented in Fig. 3. Numerous differences were found. Therefore, for simplification purposes, only the significant treatment differences for families and genera with a relative abundance of > 1% in at least one treatment group are presented. Differentially abundant taxa on day 2 and 5 after birth are presented in Supplementary Table S7. On day 2 after birth, piglets from GEST5 sows had lower abundance of *Escherichia/Shigella* and higher abundance of the *Fusobacterium* and *Actinobacillus* genera compared to piglets from CON sows (*P *< 0.05). On day 5 after birth, piglets from GEST5 sows had higher abundance of *Rikenellaceae*_RC9_gut_group and the *Lachnospiraceae* family compared to piglets from CON sows (*P *< 0.05). Differentially abundant taxa are presented in Supplementary Table S8 for the remaining sampling time points. On day 12 after birth, there were 11 differentially abundant taxa (9 genera and 2 families) and on day 26 after birth, there was one differentially abundant genus. On day 35 after birth (day 7 pw), there were 13 differentially abundant taxa (12 genera and 1 family) and on day 69 after birth (day 41 pw), there was one differentially abundant genus. The treatment differences for the differentially abundant taxa from day 12 to day 69 after birth (day 41 pw) are presented in Supplementary Table S8.

### SCFA in sow faeces

The effect of sow feeding treatment on the SCFA profile of sow faeces on day 5 post-farrowing in CH is presented in Supplementary Table S9. Faecal concentrations of total SCFA, acetate, propionate, and butyrate were higher in GEST5 compared to CON sows (*P *< 0.05). The concentration of valerate tended to be higher (*P *= 0.05) and isovalerate was lower (*P *< 0.05) in GEST5 than CON sows. The concentration of isobutyrate did not differ between GEST5 and CON sows (*P *> 0.05). The proportion (%) of butyrate was higher (*P *< 0.05), whereas that of isobutyrate and isovalerate were lower (*P *< 0.05) in GEST5 compared to CON sows. The proportions of acetate, propionate and valerate did not differ between GEST5 and CON sows (*P *> 0.05).

## Discussion

This study investigated the effect of a sow lactation feeding strategy and a piglet creep feeding strategy on feed intake, growth performance, selected health parameters and faecal microbiota in pre- and pw pigs. The novelty of this study is that it combines sow and piglet feeding strategies aimed at increasing pw piglet intake and growth in two production settings.

The higher ADFI of GEST5 sows found during the first 5 days of lactation resulted from feeding the lower DE density gestation diet, compared to that of the lactation diet, and because all sows were curve-fed to a pre-determined daily energy allocation during lactation. The GEST5 sows started with a feed allocation of 4.4 kg per day on day 1 post-farrowing, gradually increasing to 6.7 kg per day on day 5 post-farrowing, while the CON sows started with 3.9 kg per day on day 1 post-farrowing, gradually increasing to 5.9 kg per day on day 5 post-farrowing. Furthermore, due to the differences in the chemical composition of the gestation and lactation diets, the crude protein, standard ileal digestible lysine, and fat intakes of the GEST5 sows were reduced during this period, while their crude fiber intake was increased. These dietary differences mediated the treatment-related differences in nutrient composition of the sows’ milk, as well as differences in the SCFA profile of sows’ milk and faeces. On day 5 post-farrowing, GEST5 sows’ milk had significantly lower fat in CH and numerically lower fat in IE compared to CON sows. Additionally, butyrate concentrations in milk were lower on both farms, with formate and acetate also reduced at CH. The lower soya oil inclusion in the gestation diet compared to the lactation diet likely explains these effects, as sow milk fat quantity and composition are strongly influenced by dietary fat content (Farmer and Quesnel 2009; Tanghe and De Smet 2013; Lauridsen 2020). The mechanism by which this occurs has been reviewed by Zhang et al. (2018). In brief, fatty acids in the blood plasma enter the mammary glands, and are converted to triacylglycerol molecules to form milk fat globules in sow milk (Zhang et al. 2018). The reduced fat content possibly reduced the solids percentage in GEST5 sows’ milk in CH (Hurley 2015). Additionally, in CH, GEST5 sows had increased total SCFA, acetate, propionate, butyrate and valerate concentrations in the faeces, which can be attributed to increased fiber fermentation from the higher fiber intake (from inclusion of soya hulls in the gestation diet) compared to the lactation diet (Makki et al. 2018). This corresponds with the observation of increased abundance of fiber-fermenting bacteria in the faeces of GEST5 sows in IE, as discussed below. However, the increased faecal SCFA did not result in higher SCFA concentrations in sow milk. This suggests that SCFA produced by microbial fermentation in the intestine were either not absorbed or if absorbed, were likely utilised for the sow’s energy requirements and not transferred to the milk. Furthermore, GEST5 sows had decreased faecal isobutyrate and isovalerate concentrations, most likely due to lower crude protein intake, as these SCFA are produced from protein fermentation (Jha and Berrocoso 2016).

Despite these changes in sow nutrient intake, milk composition, milk and faecal SCFA profile, there was no effect of sow feeding on BW or backfat depth changes in sows, or growth performance in suckling piglets. Moreover, there was no residual effect of GEST5 on feed, energy or nutrient intake of sows during the remaining lactation period on either farm. These results are contrary to our hypothesis that feeding sows with a gestation diet and not a lactation diet for 5 days post-farrowing would increase lactation feed intake during the subsequent lactation period. One important outcome from this study was that the GEST5 sows increased their intake of the lower energy and less nutrient-dense gestation diet to achieve the daily energy allocation of the feeding curve during early lactation. This suggests that daily feed allocations on sow lactation feeding curves could be increased during early lactation, thereby encouraging higher feed intake and hence energy and nutrient intake during this critical period and potentially increasing the voluntary feed intake of sows later in lactation.

Although statistical comparisons were not performed, the effect of the creep feeding regime appeared to vary between the farms. In CH, LMR+S-fed pigs had higher DM disappearance than DPS-fed pigs, while at IE the opposite was the case. Regardless, no differences in pre- or pw growth resulted from creep feeding treatment on either farm. The differences in DM disappearance between CH and IE may be explained by the differences in litter size, and housing conditions between farms. At CH, creep feed intake was very low (82 ± 24 g/pig during the entire lactation period) across both piglet treatments, but particularly so for DPS-fed pigs. At this level of intake, it is unlikely that a treatment effect on growth would be observed, particularly since English et al. (1980) suggested that a creep feed intake of ∼600 g/pig during the entire lactation period would be necessary to provide distinct pre- and pw growth benefits. The smaller litter size in CH might help to explain the lower creep feed intake there. This is because creep feed intake is typically higher in large litters (Klindt 2003) where competition for available milk is high. These difference in creep feed intake and litter size, together with the considerable difference in piglet weighing protocols between farms, were the main reasons for presenting each farm’s data separately in this study.

In IE, although DPS disappearance was high (∼573 g/pig) during the entire lactation period, DPS-fed pigs had similar growth to LMR+S-fed pigs. Although not measured in this study, it was speculated that creep feed wastage was considerable when DPS was manually provided in IE. The fully slatted floors in IE likely contributed to this higher feed wastage, as feed rooted from the feed trough was lost through the slats, with the wastage not being visible to the stockperson. Despite efforts to minimise feed wastage by manually feeding on a little-and-often basis, feed wastage can be a feature of creep feeding DPS (Sulabo et al. 2010b; Tokach et al. 2020). Feeding LMR+S via the automatic delivery system most likely involved much lower feed wastage, as each trough was sensor-checked frequently (∼every 25 minutes) with small allocations of feed provided at each feed delivery (∼200 ml of liquid feed; ∼30 g of DM) compared to manual DPS feeding.

Although there was no difference in weight gain between the two creep feeding regimes, the difference between both in feed disappearance is economically important. A simple economic analysis using only creep feed disappearance data showed that feed cost for DPS was ∼€0.60/pig and for LMR+S was ∼€0.46/pig at IE. When the weight gain between day 5 to weaning is accounted for, the cost per kg gain for DPS was €0.10/kg and for LMR+S €0.07/kg. This suggests that, despite the higher cost of milk replacer, feeding LMR+S in this study was more economical than feeding DPS, probably because the automatic liquid feed delivery system was more efficient at minimising feed wastage than manually creep feeding DPS.

Although the sow feeding and creep feeding treatments investigated had limited effects on pig growth performance, both treatments influenced the faecal microbiota of pigs. The GEST5 increased bacterial alpha diversity in piglet faeces at day 5 post-farrowing and day 7 pw. The maternal microbiome is known to influence offspring microbiome (Nowland et al. 2021) and changes in the sows’ faecal microbiome when fed the gestation diet during lactation most likely resulted from the higher crude fiber content of this diet. Feeding LMR+S increased piglet faecal alpha diversity on day 12 post-farrowing and at day 7 pw. This most likely was due to the inclusion of milk replacer in the LMR+S strategy, as milk replacer supplementation has increased piglet faecal bacterial diversity in previous studies (Jin et al. 2020; Correa et al. 2023). More diverse microbial communities are generally considered beneficial, as they are more stable, resistant and resilient (Shade et al. 2012).

On day 5 post-farrowing, the faecal microbial community of GEST5-fed sows differed from that of CON-fed sows and had a higher abundance of *Prevotella* and *Succinivibrio.* This was most likely mediated by the higher crude fiber content of the diet fed to GEST5 sows, as *Prevotella* (Amat et al. 2020) and *Succinivibrio* (Bergamaschi et al. 2020) are fiber fermenters. This is supported by the fact that GEST5 sows in CH had higher faecal SCFA concentrations on day 5 post-farrowing, which is indicative of higher levels of fiber fermentation. However, the faecal microbiome of GEST5 and CON sows was not distinctly different for the remaining lactation period, suggesting that effects only occurred when the treatment diet was being fed and did not persist thereafter. Bacterial diversity and the relative abundances of taxa in sows’ milk were not affected by sow feeding treatment. Nevertheless, GEST5 was associated with modulations in the piglet faecal microbiome. This suggests that modulations in offspring microbiome were most likely mediated via changes in sows’ intestinal microbiome (albeit the differentially abundant taxa in the sows are not the same as those differentially abundant in the piglets) or perhaps in part due to changes in the chemical composition of the sows’ milk rather than changes in milk microbiome.

The piglet faecal microbiota composition was influenced by sow feeding treatment; for example, piglets from GEST5 sows had a higher abundance of *Fusobacterium* and *Actinobacillus* on day 2 after birth and a higher abundance of *Rikenellaceae*_RC9_gut_group and *Lachnospiraceae* on day 5 after birth compared to piglets from CON sows. *Fusobacterium* has previously been associated with diarrhoea occurrence in suckling pigs (Cheng et al. 2018) but *Lachnospiraceae* is a family of SCFA producers and has been associated with non-diarrhoeic piglets (Duo et al. 2017). *Rikenellaceae*_RC9_gut_group is an acetate producer (Holman et al. 2017) and has been associated with better feed efficiency (Quan et al. 2018). However, in the current study, no differences in diarrhoea prevalence or feed efficiency were observed between piglets from GEST5 and CON. Interestingly, on day 2 after birth, piglets from GEST5 sows had lower *Escherichia/Shigella* abundance but by day 12 after birth, higher *Escherichia/Shigella* was observed in these piglets compared to piglets from CON sows. The *Escherichia/Shigella* group includes many bacterial species that cannot be distinguished using 16S rRNA gene sequencing (Khot and Fisher 2013). While a basic local alignment search of the differentially abundant sequence showed several matches, mainly with different *Escherichia coli* strains, in addition to *Escherichia fergusonii, Shigella sonnei*, *Shigella flexneri* and other *Escherichia* and *Shigella* spp., it is difficult to ascertain which specific genus/species/serotype was present in these pigs. Although a higher abundance of *Escherichia/Shigella* has been linked with diarrhoea (Sun et al. 2019), both the current study and a recent study from our group (Rattigan et al. 2023), observed that piglets with increased abundance of *Escherichia/Shigella* did not necessarily have a higher diarrhoea prevalence. Additionally, creep feeding also impacted the piglet faecal microbiota composition. However, the relative abundance differences of only three lowly abundant taxa (*Enterococcus*, *Roseburia* and *Akkermansia*) at day 12 after birth, can be clearly attributed to the creep feeding treatment. Overall, while both sow feeding and creep feeding treatments influenced the faecal microbiota of sows and piglets, respectively, these modifications were not sufficient to impact growth performance or diarrhoea prevalence in the piglets.

In conclusion, feeding a gestation diet to sows for 5 days post-farrowing did not increase overall lactation feed intake. Although feeding the gestation diet for the first 5 days of lactation reduced the fat content and SCFA composition of milk and increased the SCFA concentration and abundance of *Prevotella* and *Succinivibrio* in sow faeces on day 5 post-farrowing, these changes were not sufficient to affect sow energy intake, BW, backfat depth changes or piglet growth. Creep feeding a liquid mixture of milk replacer and starter diet increased feed disappearance in CH and reduced feed disappearance in IE compared to creep feeding with a dry pelleted starter diet. Growth was not affected by creep feeding strategy. Sow feeding and creep feeding treatments modulated the faecal microbiota of sows and piglets, and piglets, respectively. Nonetheless, these modifications were, for the most, part, short-lived and had little impact on piglet growth or diarrhoea prevalence.

## Disclosures

The authors declare no conflict of interest.

## Supplementary Material

txaf138_Supplementary_Data
