# Supplementary material for: Effect of feeding a gestation diet to sows for 5 days post-farrowing and feeding a liquid mixture of milk replacer and starter diet to suckling piglets on growth, selected health parameters and faecal microbiota of suckling pigs on two research farms
Source: Transl Anim Sci. 2025 Oct 9;9:txaf138. doi: 10.1093/tas/txaf138 (PMC12700651; doi:10.1093/tas/txaf138)
Supplement: txaf138_Supplementary_Data [file txaf138_supplementary_data.docx]

Effect of feeding a gestation diet to sows for 5 days post-farrowing and feeding a liquid mixture of milk replacer and starter diet to suckling piglets on growth, selected health parameters and faecal microbiota of suckling piglets on two research farms

Shiv R. Vasa^*,§^, Marion Girard^†^, Gillian E. Gardiner^§^, Paul Cormican^#^, Giuseppe Bee^†^, Keelin O’Driscoll^*^, Peadar G. Lawlor^*^.

* Teagasc, Pig Development Department, Animal and Grassland Research and Innovation Centre, Moorepark, Fermoy, Co. Cork P61 C996, Ireland

^§^ Eco-Innovation Research Centre, Department of Science, South East Technological University, Waterford City, Co. Waterford X91 K0EK, Ireland

^†^ Swine Research Group, Agroscope, Posieux 1725, Switzerland

*^#^* Teagasc, Animal and Grassland Research and Innovation Centre, Teagasc, Grange, Dunsany, Co. Meath, Ireland

Corresponding author: [Peadar.Lawlor@teagasc.ie](mailto:Peadar.Lawlor@teagasc.ie)

1. Supplementary Materials and Methods

1.1 Housing

Ireland (IE)

In IE, the sows were confined in farrowing crates (BigDutchman; Vechta, Germany) in individual farrowing pens (dimension: 2.5 m x 1.8 m) from day 109 of gestation until weaning. Sows and suckling piglets were provided with ad libitum water access from a single-bite drinker in the sow feed trough and from a bowl drinker in the farrowing pens, respectively. The sow lying area in the farrowing pens had a cast iron slatted floor and the remaining farrowing pen had plastic slatted floor with a water-heated floor pad for the piglets (BigDutchman). Environmental enrichment in the farrowing room was provided via a jute bag tied to the front of the crate for the sow and a star-shaped plastic toy (Easyfix Luna 142, Easyfix, Galway, Ireland) attached to a metal chain mounted on the side of the pen for the piglets. In all of the rooms, temperature was automatically controlled and the ventilation was provided by punched ceiling ventilation with air exhausted by a variable speed fan (Big Dutchman 135). The temperature in the farrowing rooms was maintained at ~24 °C during farrowing and was gradually reduced to 22.5 °C by day 7 of lactation. The heat pads for the piglets were maintained at a temperature of 35-38 °C during lactation. At weaning, piglets were transferred to weaner pens (2.5 × 2.0 m) with fully slatted plastic floors. Ad libitum feed and water access in weaner pens was provided with a shelf-type single-space (33cm) wet-dry feeder with inset nipple drinker and an additional bowl water drinker was provided in each pen. Enrichment was provided by two star-shaped plastic toys (Easyfix Luna 142, Easyfix) and a chain mounted on the side of the pens. Temperature of the weaner rooms was maintained at 28 °C in the first week after weaning and was reduced by 2°C each week to 22°C at the end of 4 weeks post-weaning (pw).

Switzerland (CH)

In CH, the sows were loose housed in individual farrowing pens (3.29 × 2.16 m) from day 105 of gestation. The farrowing pens consisted of a concrete solid floor (2.73 × 2.16 m) and a concrete slatted floor (0.56 × 2.16 m) which included a heat covered area for the piglets. Sows and suckling piglets were provided with *ad libitum* water access from a nipple drinker attached to the wall of the pen. A small quantity of straw was provided on the floor everyday as enrichment in farrowing pens, as required by Swiss legislation. The temperature of the farrowing rooms was maintained at 24 °C and the temperature of the heat covered area of the piglets was set at 40 °C at birth and was gradually decreased by 0.5 °C each day to reach a final temperature of 32 °C on day 16 after birth. At weaning, piglets were transferred to weaner pens of (2.85 × 1.75 m) with a concrete solid floor (1.85 × 1.75 m) and a concrete slatted floor (1.00 × 1.75 m). *Ad libitum* feed and water access in weaner pens was provided with a 50x50x20 cm only dry feeder trough and a separate nipple drinker. A small quantity of straw was provided on the floor in weaner pens as enrichment. Temperature of the weaner rooms was maintained at 24 °C.

1.2 Pre-weaning liquid feeding system

Every morning at ~06:00h, the system was cleaned in closed circuit, including the mixing tanks and the pipelines with a 1% acid solution (Deosan Acidbrite AG313, Diversey Europe Operations BV, Utrecht, The Netherlands). Additionally, the system was cleaned once a week with a 0.5% solution of an alkaline detergent (AvalKsan, Gold Standard CF, Carbon Group, Ringaskiddy, Ireland), in order to remove lime scale from the circuit. In IE, after cleaning the system every morning (~08.30h), the feed troughs were also cleaned with air pressure and rinsed with acid solution daily and with alkaline solution once a week. In CH, the feed troughs were manually cleaned with water and acid/alkaline solution every morning. On both farms, before cleaning of the troughs, any remaining uneaten feed was quantified in each trough.

1.3 Sow body weight and backfat depth

The empty farrowing weight of sows was calculated as described in NRC (1998):

Empty farrowing weight = [Sow weight at day 110 of gestation– (total number of piglets born × 2.25)].

The value of 2.25 kg is an estimate of the increased weight in the gravid uterus and in mammary tissue attributed to each pig in a litter. In both IE and CH, the sow backfat depth was measured using a digital ultrasound indicator (Renco Lean-Meater, Renco Corporation, Minnesota, USA). The probe was placed on the back of the sow on each side at the level of the last rib, 6 cm from the side of the backbone. The right and left side readings from each sow were averaged and recorded. The change in backfat depth from day 109 of gestation to weaning and from weaning to service was calculated.

1.4 Visual scoring of faecal consistency

Visual scoring of faecal consistency at pen level was performed on day 12 and 20 after birth, at weaning and on day 2, 4, 6, 8, 10 and 12 pw. A 4-grade scoring system (Casey et al., 2007) was used as follows: 0 for dry pelleted faeces; 1 for soft faeces with shape; 2 for mild diarrhoea (very soft without shape or viscous liquid faeces) and 3 for severe diarrhoea (watery or with blood). Pen level scores were determined by recording the faecal score of five pigs per pen and then averaging the scores. A faecal score of 2 or 3 was considered as indicative of diarrhoea. Diarrhoea prevalence was calculated by dividing the number of faecal scores of 2 or 3 by the total number of faecal scores recorded for the pre-weaning period (day 12 to weaning) or the pw period (day 2 to 12 pw).

1.5 Short chain fatty acid analysis of sow faeces

The short chain fatty acid profile in the sow faeces was determined by high-performance liquid chromatography. Briefly, faecal samples were weighed after collection and were stored in a 50 ml tube with 1 ml of phosphoric acid (25%, w/v) at −20 °C. Following defrosting, 1 ml of internal standard (pivalic acid at 1%, w/v) and 18 ml of distilled water were added into the tube. This solution was stirred for 3 hours at room temperature and was then centrifuged for 5 minutes at 4000 g. The supernatants were filtered and analysed short chain fatty acid concentration using high-performance liquid chromatography (Ultimate 3000, Thermo Fisher Scientific, Reinach, Switzerland) with an exchange ion column (Nucleogel ION 300 OA 300 × 7.8 mm, Marcherey-Nagel AG, Oensingen, Switzerland) and equipped with a refractive index detector (RefractoMax 521, Thermo Fisher Scientific, Reinach, Switzerland).

1. Supplementary results

2.1 Mortality

There was no sow feeding × creep feeding interaction effect or any associated main effect on the percentage of mortality between 48 hours after birth and weaning in either IE or CH (P > 0.05). In IE, the percentage of mortality during this period was 12.2%, 13.1%, 17.2% and 11.7% (SEM=4.5) for litters from CON sows creep fed with DPS, litters from CON sows creep fed with LMR+S, litters from GEST5 sows creep fed with DPS and litters from GEST5 sows creep fed with LMR+S, respectively. In CH, the percentage of mortality was 3.0%, 7.3%, 2.9% and 2.5% (SEM=1.7) for litters from CON sows creep fed with DPS, litters from CON sows creep fed with LMR+S, litters from GEST5 sows creep fed with DPS and litters from GEST5 sows creep fed with LMR+S, respectively. The causes of deaths during the per-weaning period included starvation, crushing, leg injuries and meningitis. There were not enough deaths/removals in the pw postweaning to conduct statistical analysis. In IE, between weaning and day 43 pw, only 2 pigs originating from GEST5 sows creep fed with LMR+S were removed due to tail biting and 1 pig originating from CON sows creep fed with LMR+S had sudden death. In CH, between weaning and day 14 pw, only 1 pig originating from CON sows creep fed with LMR+S died due to leg injury and 1 pig originating from CON sows creep fed with DPS died due to severe diarrhoea.

2.2 Average daily dry matter disappearance and CV of individual piglet BW within litter

The effect of sow feeding and creep feeding regimes and their associated interactions on the IE and CH research farms on average daily dry matter dry matter disappearance (ADDMD) and CV of BW within litter of suckling piglets are presented in Supplementary Table S5.

In IE, there was a sow feeding × creep feeding interaction effect on ADDMD in all pre-weaning time periods. From day 5-12 after birth, piglets from CON sows fed with LMR+S had higher ADDMD than piglets fed with DPS from both GEST5 and CON sows (*P* < 0.05). From day 12-20 after birth, piglets from CON sows fed with DPS had higher ADDMD than piglets fed with LMR+S from both GEST5 and CON sows (*P* < 0.05). From day 20-28 after birth, piglets from both CON and GEST5 sows fed with DPS had higher ADDMD than piglets fed with LMR+S from both GEST5 and CON sows (*P* < 0.05). While, there was no sow feeding main effect (*P* > 0.05), there was a creep feeding main effect on ADDMD in all pre-weaning time periods. From day 5-12 after birth, LMR+S fed piglets had higher ADDMD than DPS fed piglets (12.7 vs 3.7 g/day; SEM=1.7; *P* < 0.05). However, DPS fed piglets had higher ADDMD than LMR+S fed piglets from day 12-20 after birth (15.3 vs 9.6 g/day; SEM=1.3; *P* < 0.05) and from day 20-28 after birth (63.4 vs 15.6 g/day; SEM=6.1; *P* < 0.05). In IE, there was no sow feeding × creep feeding interaction effect or any associated main effect on CV of individual piglet BW within litter from day 0 to weaning (*P* > 0.05).

In CH, there was a sow feeding × creep feeding interaction effect on ADDMD from day 5-12 after birth, where piglets from GEST5 sows fed with LMR+S had higher ADDMD than piglets fed with DPS from both GEST5 and CON sows (*P* < 0.05). In the same time period, piglets from CON sows fed with LMR+S had higher ADDMD than piglets from CON sows fed with DPS (*P* < 0.05). From day 12-19 after birth, there was a tendency of sow feeding × creep feeding interaction, where LMR+S-fed piglets from both GEST5 and CON sows had higher ADDMD than DPS-fed piglets from GEST5 sows (*P* = 0.07). From day 19-25 after birth, there was no impact of sow feeding × creep feeding interaction on ADDMD of piglets. While, there was no sow feeding main effect (*P* > 0.05), there was a creep feeding main effect on ADDMD in all pre-weaning time periods. The LMR+S-fed piglets had higher ADDMD than DPS-fed piglets from day 5-12 after birth (4.5 vs 0.7 g/day; SEM=0.43; *P* < 0.05) and from day 12-19 after birth (10.5 vs 4.5 g/day; SEM=1.70; *P* < 0.05). From day 19-25 after birth, LMR+S-fed piglets tended to have a higher ADDMD than DPS-fed piglets (4.9 vs 2.3 g/day; SEM=0.95; *P* = 0.07). In CH, there was no effect of sow feeding × creep feeding interaction effect or any associated main effect on CV of individual piglet BW within litter from day 0 to weaning (*P* > 0.05), except for a tendency of creep feeding main effect observed on day 25 after birth. Litters fed with LMR+S tended to have lower CV of individual piglet BW within litter than DPS-fed litters on day 25 after birth (13.4 vs 17.4 %; SEM=1.34; *P* = 0.05).

Supplementary Table S1 Analysed chemical composition of the experimental diets used on the Irish research farm (on an air-dry basis; g/kg unless otherwise stated)

| Analysed chemical composition | Diet 1: Gestation | Diet 2: Lactation | Diet 3: Starter | Diet 4:  Link | Diet 5:  Weaner |
| --- | --- | --- | --- | --- | --- |
| Dry matter | 884.0 | 887.9 | 920.8 | 899.0 | 876.0 |
| Crude protein | 117.0 | 162.0 | 188 | 184.0 | 172.0 |
| Ash | 42.1 | 45.4 | 52.8 | 50.0 | 38.0 |
| Crude fat | 38.7 | 68.7 | 106.0 | 63.4 | 46.8 |
| Crude fiber | 82.7 | 31.5 | 18.5 | 20.0 | 26.0 |
| Neutral detergent fiber | 198.0 | 104.0 | 60.3 | 80.7 | 126.3 |
| Acid detergent fiber | 99.3 | 46.0 | 31.0 | 25.9 | 39.1 |

Supplementary Table S2 Analysed chemical composition of the experimental diets used on the Swiss research farm (on an air-dry basis; g/kg unless otherwise stated)

| Analysed chemical composition | Diet 1: Gestation | Diet 2: Lactation | Diet 3: Starter |
| --- | --- | --- | --- |
| Dry matter | 896.3 | 902.5 | 938.7 |
| Crude protein | 122.8 | 189.3 | 205.8 |
| Ash | 44.5 | 46.0 | 60.7 |
| Crude fat | 32.2 | 68.9 | 129.0 |
| Crude fiber | 93.2 | 31.1 | 19.8 |
| Neutral detergent fiber | 228.3 | 133.8 | 69.9 |
| Acid detergent fiber | 115.8 | 50.2 | 27.2 |

Supplementary Table S3 Effect of sow feeding treatment (CON or GEST5) on sow daily digestible energy, crude protein, lysine, crude fiber, crude fat intake from farrowing to weaning on the Irish (IE) and Swiss (CH) research farms [Least square means ± pooled standard errors of the mean (SEM)].

| IE | | | | |
| --- | --- | --- | --- | --- |
| Sow feeding | CON | GEST5 | SEM | *P value* |
| Number of sows/litters | 13 | 14 |  |  |
| Average daily digestible energy intake1 (MJ/sow/day) |  |  |  |  |
| Day 0-5 | 79 | 79 | 2.8 | 0.74 |
| Day 6-14 | 116 | 109 | 4.0 | 0.30 |
| Day 15-weaning3 | 131 | 125 | 3.5 | 0.35 |
| Overall | 106 | 103 | 2.9 | 0.44 |
|  |  |  |  |  |
| Average daily net energy intake1 (MJ/sow/day) |  |  |  |  |
| Day 0-5 | 55 | 52 | 1.9 | 0.45 |
| Day 6-14 | 81 | 77 | 2.9 | 0.27 |
| Day 15-weaning3 | 92 | 88 | 2.6 | 0.34 |
| Overall | 79 | 73 | 2.1 | 0.68 |
|  |  |  |  |  |
| Average daily crude protein intake2 (g/sow/day) |  |  |  |  |
| Day 0-5 | 826 | 685 | 27 | 0.02 |
| Day 6-14 | 1123 | 1147 | 48 | 0.22 |
| Day 15-weaning^3^ | 1394 | 1332 | 43 | 0.33 |
| Overall | 1206 | 1122 | 34 | 0.10 |
|  |  |  |  |  |
| Average daily standard ileal digestible lysine intake1 (g/sow/day) |  |  |  |  |
| Day 0-5 | 55 | 39 | 1.7 | <0.01 |
| Day 6-14 | 82 | 76 | 3.2 | 0.19 |
| Day 15-weaning^3^ | 93 | 89 | 2.9 | 0.33 |
| Overall | 80 | 73 | 2.3 | 0.05 |
|  |  |  |  |  |
| Average daily crude fiber intake2 (g/sow/day) |  |  |  |  |
| Day 0-5 | 161 | 484 | 15 | <0.01 |
| Day 6-14 | 239 | 249 | 11 | 0.35 |
| Day 15-weaning^3^ | 271 | 259 | 6 | 0.38 |
| Overall | 209 | 327 | 10 | <0.01 |
|  |  |  |  |  |
| Average daily crude fat intake2 (g/sow/day) |  |  |  |  |
| Day 0-5 | 350 | 227 | 8 | <0.01 |
| Day 6-14 | 522 | 481 | 17 | 0.11 |
| Day 15-weaning3 | 591 | 565 | 16 | 0.31 |
| Overall | 450 | 374 | 12 | <0.01 |
| CH | | | | |
| Sow feeding | CON | GEST5 | SEM | *P value* |
| Number of sows/litters | 11 | 12 |  |  |
| Average daily digestible energy intake1 (MJ/sow/day) |  |  |  |  |
| Day 0-5 | 70 | 73 | 3.3 | 0.53 |
| Day 6-14 | 108 | 107 | 2.5 | 0.69 |
| Day 15-weaning3 | 114 | 118 | 4.3 | 0.56 |
| Overall | 97 | 99 | 2.8 | 0.67 |
|  |  |  |  |  |
| Average daily net energy intake1 (MJ/sow/day) |  |  |  |  |
| Day 0-5 | 50 | 50 | 2.3 | 0.99 |
| Day 6-14 | 64 | 64 | 1.5 | 0.71 |
| Day 15-weaning3 | 68 | 70 | 2.6 | 0.56 |
| Overall | 61 | 61 | 1.8 | 0.54 |
|  |  |  |  |  |
| Average daily crude protein intake2 (g/sow/day) |  |  |  |  |
| Day 0-5 | 880 | 724 | 36 | 0.31 |
| Day 6-14 | 1364 | 1344 | 28 | 0.68 |
| Day 15-weaning3 | 1441 | 1487 | 47 | 0.58 |
| Overall | 1104 | 1093 | 31 | 0.81 |
|  |  |  |  |  |
| Average daily standard ileal digestible lysine intake1 (g/sow/day) |  |  |  |  |
| Day 0-5 | 50 | 41 | 2.5 | 0.08 |
| Day 6-14 | 78 | 77 | 1.9 | 0.69 |
| Day 15-weaning3 | 83 | 85 | 3.2 | 0.56 |
| Overall | 73 | 71 | 2.1 | 0.58 |
|  |  |  |  |  |
| Average daily crude fiber intake2 (g/sow/day) |  |  |  |  |
| Day 0-5 | 145 | 439 | 12 | <0.01 |
| Day 6-14 | 224 | 221 | 5 | 0.73 |
| Day 15-weaning3 | 238 | 245 | 12 | 0.76 |
| Overall | 174 | 263 | 8 | <0.01 |
|  |  |  |  |  |
| Average daily crude fat intake2 (g/sow/day) |  |  |  |  |
| Day 0-5 | 320 | 203 | 12 | <0.01 |
| Day 6-14 | 497 | 451 | 11 | 0.67 |
| Day 15-weaning3 | 527 | 512 | 18 | 0.57 |
| Overall | 413 | 378 | 11 | 0.04 |

Abbreviations: CON = sows fed with a lactation diet from one day post-farrowing until weaning; GEST5 = sows fed with a gestation diet for 5 days post-farrowing followed by a lactation diet until weaning; IE = research farm in Ireland; CH = research farm in Switzerland.

1 Calculated chemical composition values.

2 Analysed chemical composition values.

3 Weaning in IE = 29.0 ± 0.1 day of lactation; Weaning in CH = 25.5 ± 1.3 day of lactation.

Supplementary Table S4 Effect of sow feeding treatment (CON or GEST5), creep feeding treatment (DPS or LMR+S) and their associated interactions on body weight and backfat depth of sows from day 107 of gestation to service on the Irish (IE) and Swiss (CH) research farms [Least square means ± pooled standard errors of the mean (SEM)].

| IE | | | | | | | | | |
| --- | --- | --- | --- | --- | --- | --- | --- | --- | --- |
| Sow feeding | CON | | | GEST5 | | SEM | *P-*value | | |
| Creep feeding | DPS | | LMR+S | DPS | LMR+S |  | *Sow feed* | *Creep feed* | *Sow feed × Creep feed* |
| Number of sows | 7 | | 6 | 7 | 7 |  |  |  |  |
| BW (kg) |  | |  |  |  |  |  |  |  |
| Day 108 of gestation | 268 | | 296 | 275 | 283 | 17.7 | 0.96 | 0.66 | 0.92 |
| Post-farrowing1 | 234 | | 251 | 230 | 245 | 17.1 | 0.47 | 0.29 | 0.71 |
| Weaning2 | 235 | | 266 | 246 | 246 | 19.1 | 0.96 | 0.79 | 0.95 |
| Service | 222 | | 241 | 197 | 233 | 17.3 | 0.52 | 0.23 | 0.50 |
| Overall | 245 | | 261 | 243 | 259 | 17.2 | 0.82 | 0.14 | 0.99 |
|  |  | |  |  |  |  |  |  |  |
| Change in bodyweight from day 108 of gestation to post-farrowing3 | -34.4 | | -44.6 | -45.6 | -37.3 | 3.59 | 0.53 | 0.23 | 0.11 |
| Change in bodyweight from post-farrowing to weaning4 | 1.6 | | 15.3 | 16.3 | 0.7 | 1.32 | 0.22 | 0.45 | 0.22 |
| Change in bodyweight from post-weaning to service5 | -13.1 | | -24.5 | -48.3 | -12.3 | 12.4 | 0.38 | 0.35 | 0.64 |
|  |  | |  |  |  |  |  |  |  |
| Backfat depth (mm) |  | |  |  |  |  |  |  |  |
| Day 108 of gestation | 16.6 | | 15.4 | 15.2 | 15.3 | 1.50 | 0.70 | 0.67 | 0.89 |
| Weaning2 | 12.7 | | 12.6 | 12.5 | 12.6 | 1.28 | 0.99 | 0.92 | 1.00 |
| Service | 12.2 | | 11.9 | 11.6 | 12.1 | 1.16 | 0.98 | 0.95 | 0.98 |
| Overall | 13.9 | | 13.1 | 13.1 | 13.4 | 1.28 | 0.86 | 0.81 | 0.67 |
|  |  | |  |  |  |  |  |  |  |
| Change in backfat depth from day 108 to weaning6 | -3.93 | | -2.79 | -2.25 | -2.71 | 0.86 | 0.44 | 0.77 | 0.51 |
| Change in backfat depth from weaning to service7 | -0.83 | | -0.45 | -0.50 | -0.45 | 0.49 | 0.92 | 0.94 | 0.50 |
| CH | | | | | | | | | |
| Sow feeding | CON | | | GEST5 | | SEM | *P-*value | | |
| Creep feeding | DPS | LMR+S | | DPS | LMR+S |  | *Sow feed* | *Creep feed* | *Sow feed × Creep feed* |
| Number of sows | 6 | 5 | | 6 | 6 |  |  |  |  |
| BW (kg) |  |  | |  |  |  |  |  |  |
| Day 108 of gestation | 275 | 278 | | 289 | 279 | 5.12 | 0.18 | 0.50 | 0.25 |
| Post-farrowing1 | 260 | 255 | | 274 | 258 | 6.74 | 0.23 | 0.14 | 0.23 |
| Weaning2 | 245 | 241 | | 256 | 247 | 8.18 | 0.32 | 0.44 | 0.63 |
| Service | 230 | 228 | | 240 | 235 | 7.39 | 0.25 | 0.67 | 0.65 |
| Overall | 252 | 251 | | 265 | 254 | 6.27 | 0.96 | 0.20 | 0.58 |
|  |  |  | |  |  |  |  |  |  |
| Change in bodyweight from day 108 of gestation to post-farrowing3 | -15.2 | -23.3 | | -14.9 | -21.1 | 3.62 | 0.74 | 0.07 | 0.78 |
| Change in bodyweight from post-farrowing to weaning4 | -15.2 | -14.4 | | -18.6 | -11.0 | 5.30 | 1.00 | 0.44 | 0.53 |
| Change in bodyweight from post-weaning to service5 | -15.0 | -12.8 | | -15.6 | -11.2 | 3.45 | 0.88 | 0.35 | 0.75 |
|  |  |  | |  |  |  |  |  |  |
| Backfat depth (mm) |  |  | |  |  |  |  |  |  |
| Day 108 of gestation | 15.8 | 15.7 | | 15.8 | 15.1 | 1.10 | 0.86 | 0.68 | 0.97 |
| Weaning2 | 13.1 | 13.0 | | 13.1 | 12.4 | 0.86 | 0.76 | 0.58 | 0.92 |
| Service | 12.4 | 12.2 | | 12.2 | 12.1 | 0.70 | 0.97 | 0.76 | 0.99 |
| Overall | 13.7 | 13.4 | | 13.6 | 13.2 | 0.85 | 0.85 | 0.65 | 0.92 |
|  |  |  | |  |  |  |  |  |  |
| Change in backfat depth from day 108 to weaning6 | -2.67 | -2.70 | | -2.75 | -2.75 | 0.66 | 0.92 | 0.98 | 0.98 |
| Change in backfat depth from weaning to service7 | -0.75 | -0.80 | | -0.83 | -0.25 | 0.31 | 0.46 | 0.40 | 0.32 |

Abbreviations: CON = sows fed with a lactation diet from one day post farrowing until weaning; GEST5 = sows fed with a gestation diet for 5 days post-farrowing followed by a lactation diet until weaning; DPS = suckling piglets provided with dry pelleted starter diet from day 5 to weaning; LMR+S = suckling piglets provided with a liquid mixture of milk replacer and starter diet from day 5 to weaning; IE = research farm in Ireland; CH = research farm in Switzerland.

1 Estimated value: empty farrowing weight = [sow weight at day 109 of gestation – (total born × 2.25)]. The value of 2.25 kg is an estimate of the increased weight in the gravid uterus and in mammary tissue attributed to each pig in a litter (NRC, 1998).

2 Weaning in IE = 29.0 ± 0.1 day of lactation; Weaning in CH = 25.5 ± 1.3 day of lactation.

3 Sow bodyweight change from day 109 of gestation to post-farrowing = (sow bodyweight post-farrowing – sow bodyweight at day 109 of gestation)

4 Sow bodyweight change from post-farrowing to weaning = (sow bodyweight at weaning – sow bodyweight post-farrowing)

5 Sow bodyweight change from weaning to service = (sow bodyweight at service – sow bodyweight at weaning)

6 Sow backfat depth change from day 109 of gestation to weaning = (sow backfat depth at weaning – sow backfat depth at day 109 of gestation).

^7^ Sow backfat depth change from weaning to service = (sow backfat depth at service – sow backfat depth at weaning).

Supplementary Table S5 Effect of sow feeding treatment (CON or GEST5), creep feeding treatment (DPS or LMR+S) and their associated interactions on average daily dry matter disappearance, CV of individual piglet BW within litter, medication usage, clinical cases of disease and diarrhoea prevalence of suckling piglets on the Irish (IE) and Swiss (CH) research farms [Least square means ± pooled standard errors of the mean (SEM)].

| IE | | | | | | | | |
| --- | --- | --- | --- | --- | --- | --- | --- | --- |
| Sow feeding | CON | | GEST5 | | SEM | *P-*value | | |
| Creep feeding | DPS | LMR+S | DPS | LMR+S |  | *Sow feed* | *Creep feed* | *Sow feed × Creep feed* |
| Number of sows/litters | 7 | 6 | 7 | 7 |  |  |  |  |
| ADDMD (DM basis, g/pig/day) |  |  |  |  |  |  |  |  |
| Day 5-12 | 3.3^a^ | 16.7^b^ | 4.1^a^ | 8.6^ab^ | 2.35 | 0.14 | <0.01 | <0.01 |
| Day 12-20 | 17.5^a^ | 10.5^b^ | 13.2^ab^ | 8.8^b^ | 1.84 | 0.12 | 0.01 | 0.02 |
| Day 20-28 | 56.6^a^ | 16.8^b^ | 70.1^a^ | 14.5^b^ | 8.69 | 0.53 | <0.01 | <0.01 |
| Overall | 25.8 | 14.6 | 29.2 | 10.6 | 3.36 | 0.92 | <0.01 | 0.29 |
|  |  |  |  |  |  |  |  |  |
| CV of individual piglet BW within litter (%) |  |  |  |  |  |  |  |  |
| Day 0 | 19.0 | 25.0 | 21.1 | 16.7 | 1.59 | 0.51 | 0.46 | 0.79 |
| Day 5 | 19.0 | 22.8 | 17.4 | 17.7 | 2.57 | 0.18 | 0.34 | 0.44 |
| Day 12 | 17.1 | 23.0 | 23.2 | 15.5 | 3.63 | 0.81 | 0.90 | 0.48 |
| Day 20 | 19.1 | 21.7 | 20.8 | 16.3 | 2.76 | 0.46 | 0.83 | 0.71 |
| Day 281 | 18.5 | 22.1 | 19.5 | 15.2 | 2.50 | 0.21 | 0.99 | 0.43 |
| Overall | 19.7 | 23.5 | 20.3 | 17.6 | 2.34 | 0.28 | 0.82 | 0.19 |
|  |  |  |  |  |  |  |  |  |
| Antibiotic usage2 (ml/pig) | 0.06 | 0.10 | 0.05 | 0.06 | 0.04 | 0.48 | 0.57 | 0.64 |
| Anti-inflammatory usage2 (ml/pig) | 0.01 | 0.02 | 0.01 | 0.01 | 0.01 | 0.48 | 0.57 | 0.64 |
| Number of clinical cases / litter3 | 0.57 | 1.00 | 0.57 | 0.71 | 0.40 | 0.72 | 0.48 | 0.72 |
| Diarrhoea prevalence in pigs from day 12 to weaning4 (%) | 14.3 | 16.7 | 14.3 | 19.1 | 8.2 | 0.89 | 0.67 | 0.89 |
| CH | | | | | | | | |
| Sow feeding | CON | | GEST5 | | SEM | *P-value* | | |
| Creep feeding | DPS | LMR+S | DPS | LMR+S |  | *Sow feed* | *Creep feed* | *Sow feed × Creep feed* |
| Number of sows/litters | 6 | 5 | 6 | 6 |  |  |  |  |
| ADDMD (DM basis, g/pig/day) |  |  |  |  |  |  |  |  |
| Day 5-12 | 0.4^a^ | 4.0^bc^ | 1.1^ab^ | 4.9^c^ | 0.60 | 0.19 | <0.01 | <0.01 |
| Day 12-19 | 4.9^AB^ | 10.5^A^ | 2.5^B^ | 10.4^A^ | 2.40 | 0.62 | 0.01 | 0.07 |
| Day 19-25 | 3.4 | 5.3 | 1.3 | 4.4 | 1.34 | 0.28 | 0.07 | 0.21 |
| Overall | 2.9 | 6.6 | 1.6 | 6.6 | 1.30 | 0.16 | 0.06 | 0.85 |
|  |  |  |  |  |  |  |  |  |
| CV of individual piglet BW within litter (%) |  |  |  |  |  |  |  |  |
| Day 0 | 17.7 | 17.4 | 21.1 | 18.8 | 2.57 | 0.36 | 0.62 | 0.37 |
| Day 5 | 17.2 | 14.7 | 17.9 | 13.5 | 1.96 | 0.91 | 0.10 | 0.39 |
| Day 12 | 16.1 | 12.8 | 17.3 | 14.3 | 1.80 | 0.47 | 0.11 | 0.22 |
| Day 19 | 16.8 | 11.2 | 16.2 | 15.4 | 1.87 | 0.33 | 0.11 | 0.10 |
| Day 251 | 17.9 | 10.9 | 16.8 | 15.8 | 1.88 | 0.33 | 0.05 | 0.10 |
| Overall | 17.2 | 13.4 | 17.9 | 15.6 | 1.72 | 0.41 | 0.11 | 0.68 |
|  |  |  |  |  |  |  |  |  |
| Antibiotic usage2 (ml/pig) | 0.04 | 0.01 | 0.02 | 0.02 | 0.03 | 0.77 | 0.50 | 0.42 |
| Anti-inflammatory usage2 (ml/pig) | 0.01 | 0.00 | 0.00 | 0.01 | 0.01 | 0.70 | 0.70 | 0.21 |
| Number of clinical cases of disease / litter3 | 0.33 | 0.20 | 0.17 | 0.83 | 0.30 | 0.45 | 0.39 | 0.20 |
| Diarrhoea prevalence from day 12 to weaning4 (%) | 11.1 | 6.7 | 16.7 | 5.6 | 7.0 | 0.88 | 0.31 | 0.71 |

Abbreviations: CON = sows fed with a lactation diet from one day post farrowing until weaning; GEST5= sows fed with a gestation diet for 5 days post-farrowing followed by a lactation diet until weaning; DPS = suckling piglets provided with dry pelleted starter diet from day 5 to weaning; LMR+S = suckling piglets provided with a liquid mixture of milk replacer and starter diet from day 5 to weaning; IE = research farm in Ireland; CH = research farm in Switzerland; ADDMD = Average daily dry matter disappearance; DM = dry matter.

^1^ Weaning in IE = 29.0 ± 0.1 day of lactation; Weaning in CH = 25.5 ± 1.3 day of lactation.

^2^ In IE, Unicillin (Procaine Benzlypenicllin, 300 mg/ml injection, Univet, Cootehill, Cavan, Ireland) was used as the antibiotic and Loxicom 5 mg/ml injection, (Norbrook, Monaghan, Ireland) was used as the anti-inflammatory. In CH, Betamox (Norbrook) was used as the antibiotic and Rifen (Streuli Tiergesundheit AG, Uznach, Switzerland) was used as the anti-inflammatory. The values were averaged by dividing the total volume used per litter by the number of piglets in each litter.

^3^ The number of pigs in each litter that were treated on one or more occasion.

^4^ Visual scoring of faecal consistency at pen level was performed on day 12, 20 after birth and weaning using a 4-grade scoring system (Casey et al., 2007) as follows: 0 for dry pelleted faeces; 1 for soft faeces with shape; 2 for mild diarrhoea (very soft without shape or viscous liquid faeces) and 3 for severe diarrhoea (watery or with blood). A fecal score of 2 or greater for a pen was considered indicative of diarrhoea at each time point between day 12 and weaning. The overall diarrhoea prevalence was reported for the pre-weaning period.

^a-c^ Values within a row that do not share a common superscript differ significantly at *P* < 0.05.

^A-B^ Values within a row that do not share a common superscript tended to differ significantly at 0.05 < *P* <0.1.

Supplementary Table S6 Effect of sow feeding treatment (CON or GEST5), creep feeding treatment (DPS or LMR+S) and their associated interactions on feed intake, growth performance and diarrhoea prevalence of weaned piglets from day 0 to 43 post-weaning on the Irish research farm (IE) and from day 0 to 14 post-weaning on the Swiss research farm (CH) [Least square means ± pooled standard errors of the mean (SEM)].

| IE | | | | | | | | |  |
| --- | --- | --- | --- | --- | --- | --- | --- | --- | --- |
| Sow feeding | CON | | GEST5 | |  | *P-*value | | |  |
| Creep feeding | DPS | LMR+S | DPS | LMR+S | SEM | *Sow feed* | *Creep feed* | *Sow feed × Creep feed* | |
| Number of pens | 7 | 8 | 7 | 7 |  |  |  |  | |
| BW (kg) |  |  |  |  |  |  |  |  | |
| Day 0 (weaning^1^) | 8.9 | 9.2 | 9.0 | 8.8 | 0.40 | 0.64 | 0.64 | 0.64 | |
| Day 7 | 10.6 | 10.6 | 10.6 | 10.3 | 0.14 | 0.39 | 0.33 | 0.32 | |
| Day 14 | 13.1 | 13.2 | 13.0 | 12.4 | 0.27 | 0.13 | 0.28 | 0.18 | |
| Day 21 | 17.0 | 16.5 | 16.6 | 16.1 | 0.35 | 0.23 | 0.14 | 0.32 | |
| Day 43 | 34.4 | 34.0 | 33.7 | 33.3 | 0.76 | 0.40 | 0.58 | 0.79 | |
| Overall | 18.8 | 18.6 | 18.5 | 18.0 | 0.33 | 0.23 | 0.30 | 0.70 | |
|  |  |  |  |  |  |  |  |  | |
| ADFI (g/pig/day) |  |  |  |  |  |  |  |  | |
| Day 0-7 | 244 | 266 | 272 | 233 | 16.7 | 0.87 | 0.60 | 0.32 | |
| Day 7-14 | 480 | 477 | 488 | 406 | 21.3 | 0.17 | 0.06 | 0.05 | |
| Day 14-21 | 687 | 672 | 650 | 604 | 34.1 | 0.14 | 0.37 | 0.38 | |
| Day 21-43 | 1252 | 1272 | 1161 | 1284 | 69.8 | 0.58 | 0.31 | 0.61 | |
| Overall | 666 | 672 | 643 | 632 | 27.4 | 0.27 | 0.93 | 0.76 | |
|  |  |  |  |  |  |  |  |  | |
| ADG (g/pig/day) |  |  |  |  |  |  |  |  | |
| Day 0-7 | 224 | 233 | 242 | 231 | 19.7 | 0.74 | 0.95 | 0.94 | |
| Day 7-14 | 358 | 343 | 359 | 316 | 27.3 | 0.66 | 0.29 | 0.66 | |
| Day 14-21 | 572 | 510 | 493 | 521 | 32.6 | 0.34 | 0.60 | 0.38 | |
| Day 21-43 | 807 | 803 | 765 | 784 | 20.9 | 0.22 | 0.71 | 0.56 | |
| Overall | 490 | 472 | 465 | 463 | 16.0 | 0.39 | 0.50 | 0.58 | |
|  |  |  |  |  |  |  |  |  | |
| Gain to feed ratio (g/g) |  |  |  |  |  |  |  |  | |
| Day 0-7 | 0.94 | 0.90 | 0.88 | 0.93 | 0.05 | 0.75 | 0.92 | 0.80 | |
| Day 7-14 | 0.82 | 0.74 | 0.72 | 0.76 | 0.05 | 0.46 | 0.62 | 0.53 | |
| Day 14-21 | 0.84 | 0.77 | 0.75 | 0.86 | 0.04 | 1.00 | 0.64 | 0.24 | |
| Day 21-43 | 0.65 | 0.64 | 0.65 | 0.66 | 0.01 | 0.64 | 0.84 | 0.94 | |
| Overall | 0.81 | 0.76 | 0.75 | 0.80 | 0.02 | 0.83 | 0.90 | 0.19 | |
|  |  |  |  |  |  |  |  |  | |
| Diarrhoea prevalence from weaning to day 14 post-weaning^2^ (%) | 38.1 | 27.8 | 19.1 | 28.6 | 6.7 | 0.18 | 0.93 | 0.14 | |
| CH | | | | | | | | | |
| Sow feed | CON | | GEST5 | | SEM | *P value* | | | |
| Creep feed | DPS | LMR+S | DPS | LMR+S |  | *Sow feed* | *Creep feed* | *Sow feed × Creep feed* | |
| Number of pens | 11 | 10 | 11 | 11 |  |  |  |  | |
| BW (kg) |  |  |  |  |  |  |  |  | |
| Day 0 (weaning^1^) | 7.7 | 8.1 | 8.0 | 7.3 | 0.42 | 0.58 | 0.71 | 0.15 | |
| Day 7 | 8.4 | 8.3 | 8.4 | 8.4 | 0.11 | 0.89 | 0.55 | 0.76 | |
| Day 14 | 10.6 | 10.8 | 10.5 | 10.3 | 0.23 | 0.26 | 0.99 | 0.63 | |
| Overall | 9.50 | 9.53 | 9.44 | 9.37 | 0.16 | 0.43 | 0.83 | 0.83 | |
|  |  |  |  |  |  |  |  |  | |
|  |  |  |  |  |  |  |  |  | |
| ADFI (g/pig/day) |  |  |  |  |  |  |  |  | |
| Day 0-7 | 165 | 156 | 159 | 162 | 9.5 | 0.98 | 0.71 | 0.91 | |
| Day 7-14 | 332 | 358 | 327 | 315 | 17.8 | 0.18 | 0.70 | 0.41 | |
| Overall | 249 | 257 | 243 | 238 | 12.5 | 0.32 | 0.89 | 0.64 | |
|  |  |  |  |  |  |  |  |  | |
| ADG (g/pig/day) |  |  |  |  |  |  |  |  | |
| Day 0-7 | 99 | 71 | 84 | 95 | 17.0 | 0.78 | 0.63 | 0.68 | |
| Day 7-14 | 308 | 352 | 301 | 283 | 24.5 | 0.11 | 0.59 | 0.27 | |
| Overall | 204 | 212 | 192 | 189 | 17.6 | 0.31 | 0.89 | 0.79 | |
|  |  |  |  |  |  |  |  |  | |
| Gain to feed ratio (g/g) |  |  |  |  |  |  |  |  | |
| Day 0-7 | 0.70 | 0.61 | 0.57 | 0.67 | 0.05 | 0.51 | 0.87 | 0.31 | |
| Day 7-14 | 0.91 | 0.93 | 0.91 | 0.90 | 0.03 | 0.72 | 0.76 | 0.93 | |
| Overall | 0.80 | 0.77 | 0.74 | 0.79 | 0.03 | 0.41 | 0.76 | 0.24 | |
|  |  |  |  |  |  |  |  |  | |
| Diarrhoea prevalence from weaning to day 14 post-weaning^2^ (%) | 66.7 | 66.7 | 80.3 | 72.7 | 5.6 | 0.80 | 0.45 | 0.45 | |

Abbreviations: CON = sows fed with a lactation diet from one day post farrowing until weaning; GEST5 = sows fed with a gestation diet for 5 days post-farrowing followed by a lactation diet until weaning; DPS = suckling piglets provided with dry pelleted starter diet from day 5 to weaning; LMR+S = suckling piglets provided with a liquid mixture of milk replacer and starter diet from day 5 to weaning; IE = research farm in Ireland; CH = research farm in Switzerland; ADG = Average daily gain; ADFI = Average daily feed intake.

^1^ Weaning in IE = 29.0 ± 0.1 day of lactation; Weaning in CH = 25.5 ± 1.3 day of lactation.

^2^ Visual scoring of faecal consistency at pen level was performed on day 2, 4, 6, 8, 10 and 12 post-weaning using a 4-grade scoring system (Casey et al., 2007) as follows: 0 for dry pelleted faeces; 1 for soft faeces with shape; 2 for mild diarrhoea (very soft without shape or viscous liquid faeces) and 3 for severe diarrhoea (watery or with blood). A fecal score of 2 or greater for a pen was considered indicative of diarrhoea at each time point between days 2 and 12 post-weaning. The overall diarrhoea prevalence was reported for the early post-weaning period.

Supplementary Table S7 Effect of sow feeding treatment (CON or GEST5) on relative abundance of bacterial taxa in piglet faeces on day 2 and day 5 after birth on the Irish research farm [Least square means ± pooled standard errors of the mean (SEM)].

| Sow feeding | CON | GEST5 | SEM | P value |
| --- | --- | --- | --- | --- |
| Number of piglets | 19 | 20 |  |  |
| Day 2 after birth |  |  |  |  |
| G-*Escherichia/Shigella* | 26.30 | 7.05 | 4.38 | <0.01 |
| G-*Fusobacterium* | 13.77 | 26.71 | 3.65 | <0.01 |
| G-*Actinobacillus* | 5.57 | 7.77 | 1.96 | <0.01 |
|  |  |  |  |  |
| Day 5 after birth |  |  |  |  |
| G-*Rikenellaceae*_RC9_gut_group | 1.77 | 7.28 | 1.92 | 0.04 |
| F-*Lachnospiraceae* | 0.97 | 2.95 | 0.78 | 0.03 |

Abbreviations: CON = sows fed with a lactation diet from one day post farrowing until weaning; GEST5 = sows fed with a gestation diet for 5 days post-farrowing followed by a lactation diet until weaning; G = genus; F = family.

Supplementary Table S8 Effect of sow feeding treatment (CON or GEST5) and creep feeding treatment (DPS or LMR+S) on relative abundance of bacterial taxa in pig faeces on day 12 and 26, 35 and 69 after birth on the Irish research farm [Least square means ± pooled standard errors of the mean (SEM)].

| Sow feeding | CON | | GEST5 | | SEM | P value^1^ |
| --- | --- | --- | --- | --- | --- | --- |
| Creep feeding | DPS | LMR+S | DPS | LMR+S |  |  |
| Number of piglets | 9 | 10 | 10 | 10 |  |  |
| Day 12 after birth |  |  |  |  |  |  |
| G-*Escherichia/Shigella* | 5.47^a^ | 5.71^ab^ | 16.46^b^ | 16.90^ab^ | 4.47 | <0.01 |
| G-*Clostridium_sensu_stricto_1* | 4.58^a^ | 1.86^b^ | 2.82^ab^ | 4.59^a^ | 1.57 | <0.01 |
| F_*Lachnospiraceae* | 0.82^a^ | 1.44^ab^ | 2.69^b^ | 2.04^ab^ | 0.62 | <0.01 |
| G-*Lactobacillus* | 0.48^a^ | 1.45^bc^ | 3.34^c^ | 1.54^b^ | 0.82 | 0.04 |
| G-*Enterococcus* | 0.30^ab^ | 0.02^a^ | 1.31^b^ | 1.00^b^ | 0.41 | 0.04 |
| G-*Roseburia* | 0.05^a^ | 1.19^b^ | 0.51^ab^ | 0.78^ab^ | 0.47 | 0.01 |
| G-*Akkermansia* | 2.05^a^ | 0.05^b^ | 1.30^ab^ | 0.67^ab^ | 0.90 | 0.01 |
| G-*Helicobacter* | 0.00^a^ | 2.07^b^ | 2.08^b^ | 0.64^b^ | 0.74 | <0.01 |
| G-*Anaerovoracaceae*_Family_XIII_AD3011_group | 0.13^a^ | 1.03^b^ | 0.67^ab^ | 0.57^ab^ | 0.21 | <0.01 |
| G-*Prevotellaceae*_NK3B31_group | 0.06^a^ | 1.02^b^ | 0.22^ab^ | 0.28^ab^ | 0.26 | 0.04 |
| F- *Erysipelotrichaceae* | 0.23^a^ | 0.32^ab^ | 1.1^b^ | 0.06^a^ | 0.01 | <0.01 |
|  |  |  |  |  |  |  |
| Day 26 after birth |  |  |  |  |  |  |
| G-*Helicobacter* | 0.17^a^ | 1.17^b^ | 1.26^b^ | 1.81^b^ | 0.38 | 0.01 |
|  |  |  |  |  |  |  |
| Day 35 after birth (day 7 post-weaning) |  |  |  |  |  |  |
| G-*Prevotella* | 24.53^ab^ | 17.60^a^ | 33.20^b^ | 31.27^b^ | 3.62 | 0.04 |
| G-*Anaerovibrio* | 3.39^a^ | 0.55^b^ | 5.20^a^ | 2.30^a^ | 1.14 | 0.01 |
| G-*Treponema* | 2.14^a^ | 7.17^b^ | 3.89^ab^ | 1.15^a^ | 1.21 | 0.01 |
| G-*Lactobacillus* | 2.79^ab^ | 1.12^a^ | 2.64^ab^ | 4.95^b^ | 1.00 | <0.01 |
| G-*Alloprevotella* | 3.20^ab^ | 2.00^a^ | 2.29^ab^ | 3.59^b^ | 0.45 | 0.02 |
| G-*Campylobacter* | 3.94^a^ | 3.51^a^ | 1.99^ab^ | 1.10^b^ | 0.93 | 0.02 |
| G-*Christensenellaceae*_R-7_group | 1.12^ab^ | 4.66^a^ | 1.88^ab^ | 0.28^b^ | 1.09 | <0.01 |
| G-*Agathobacter* | 0.87^ab^ | 0.45^a^ | 1.4^ab^ | 1.85^b^ | 0.50 | <0.01 |
| F-*UCG-010* | 0.85^ab^ | 1.83^a^ | 1.08^ab^ | 0.57^b^ | 0.33 | 0.04 |
| G-*Oscillospiraceae*-NK4A214_group | 0.66^ab^ | 1.46^a^ | 0.79^ab^ | 0.41^b^ | 0.24 | 0.01 |
| G-*Escherichia/Shigella* | 0.99^ab^ | 0.44^a^ | 0.63^a^ | 3.99^b^ | 1.34 | 0.03 |
| G-*Ruminococcus* | 1.16^ab^ | 0.72^b^ | 0.37^bc^ | 0.24^c^ | 0.36 | 0.04 |
| G-*Fusobacterium* | 0.11^ab^ | 0.00^a^ | 0.00^a^ | 2.2^b^ | 0.63 | 0.01 |
|  |  |  |  |  |  |  |
| Day 69 after birth (day 41 post-weaning) |  |  |  |  |  |  |
| G-*Treponema* | 6.85^ab^ | 13.2^a^ | 6.27^ab^ | 4.48^b^ | 3.09 | 0.03 |

Abbreviations CON = sows fed with a lactation diet from one day post farrowing until weaning; GEST5= sows fed with a gestation diet for 5 days post-farrowing followed by a lactation diet until weaning; DPS = suckling piglets provided with dry pelleted starter diet from day 5 to weaning; LMR+S = suckling piglets provided with a liquid mixture of milk replacer and starter diet from day 5 to weaning; G = genus; F = family.

1 *P* value indicates interaction effect of the sow feeding × creep feeding treatments.

a-c Values within a row that do not share a common superscript differ significantly at *P* < 0.05.

Supplementary Table S9 Effect of sow feeding treatment (CON or GEST5) on short chain fatty acid profile of sow faeces on day 5 post-farrowing on the Swiss research farm [Least square means ± pooled standard errors of the mean (SEM)].

| Sow feeding | CON | GEST5 | SEM | *P value* |
| --- | --- | --- | --- | --- |
| Number of sows | 11 | 12 |  |  |
| Total short chain fatty acids (µmol/g) | 111.3 | 161.7 | 9.56 | <0.01 |
| Acetate (µmol/g) | 65.0 | 90.0 | 5.54 | <0.01 |
| Propionate (µmol/g) | 28.1 | 37.5 | 2.98 | 0.04 |
| Butyrate (µmol/g) | 8.9 | 24.4 | 2.22 | <0.01 |
| Isobutyrate (µmol/g) | 3.0 | 2.7 | 0.17 | 0.36 |
| Valerate (µmol/g) | 2.2 | 3.6 | 0.48 | 0.05 |
| Isovalerate (µmol/g) | 4.2 | 3.5 | 0.25 | 0.04 |
|  |  |  |  |  |
| Proportion |  |  |  |  |
| Acetate (%) | 58.6 | 55.8 | 1.38 | 0.17 |
| Propionate (%) | 25.1 | 23.0 | 1.02 | 0.15 |
| Butyrate (%) | 7.7 | 14.9 | 1.14 | <0.01 |
| Isobutyrate (%) | 2.7 | 1.8 | 0.11 | <0.01 |
| Valerate (%) | 2.0 | 2.3 | 0.31 | 0.50 |
| Isovalerate (%) | 3.9 | 2.3 | 0.19 | <0.01 |

Abbreviations: CON = sows fed with a lactation diet from one day post-farrowing until weaning; GEST5 = sows fed with a gestation diet for 5 days post-farrowing followed by a lactation diet until weaning.


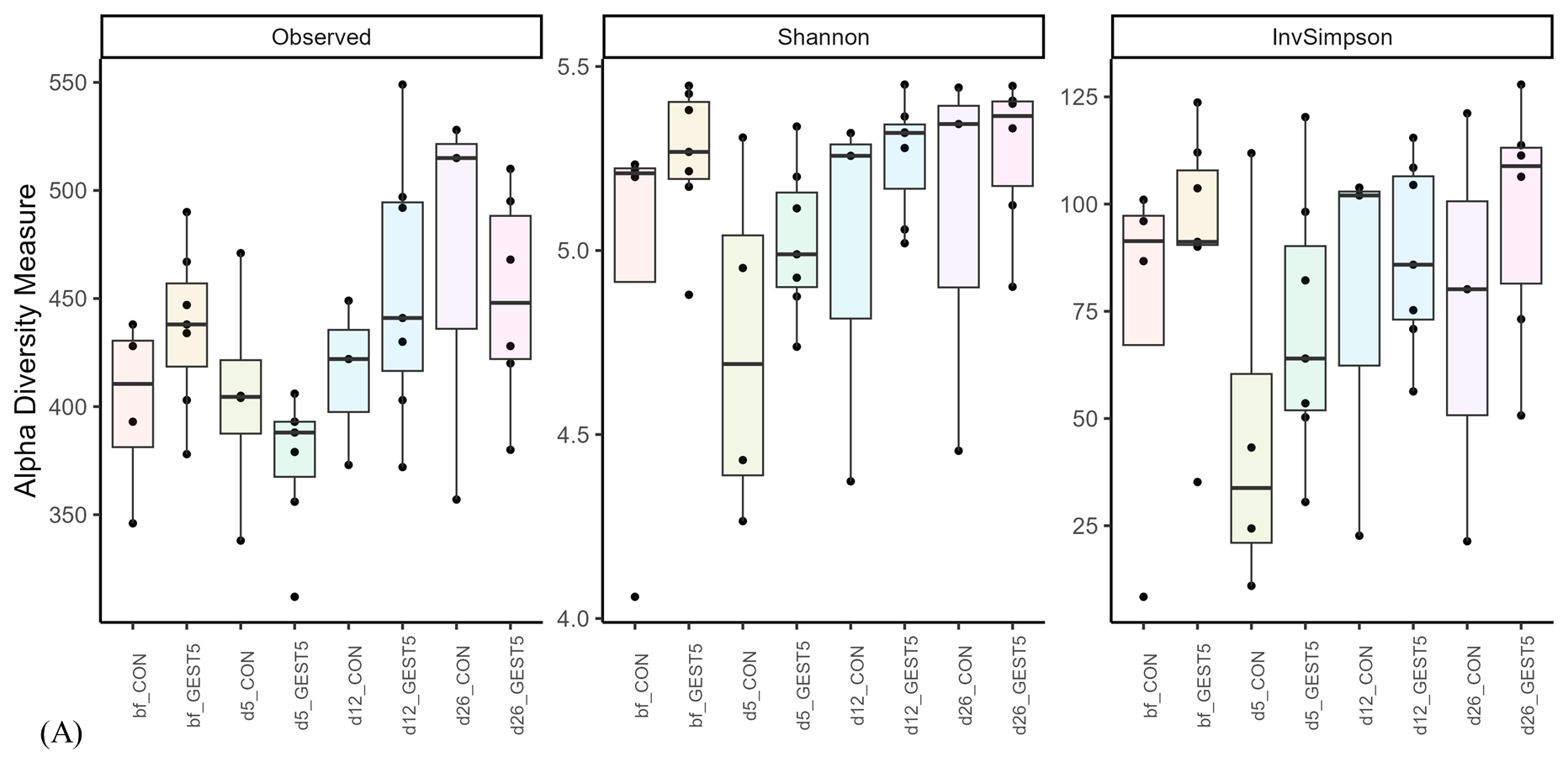


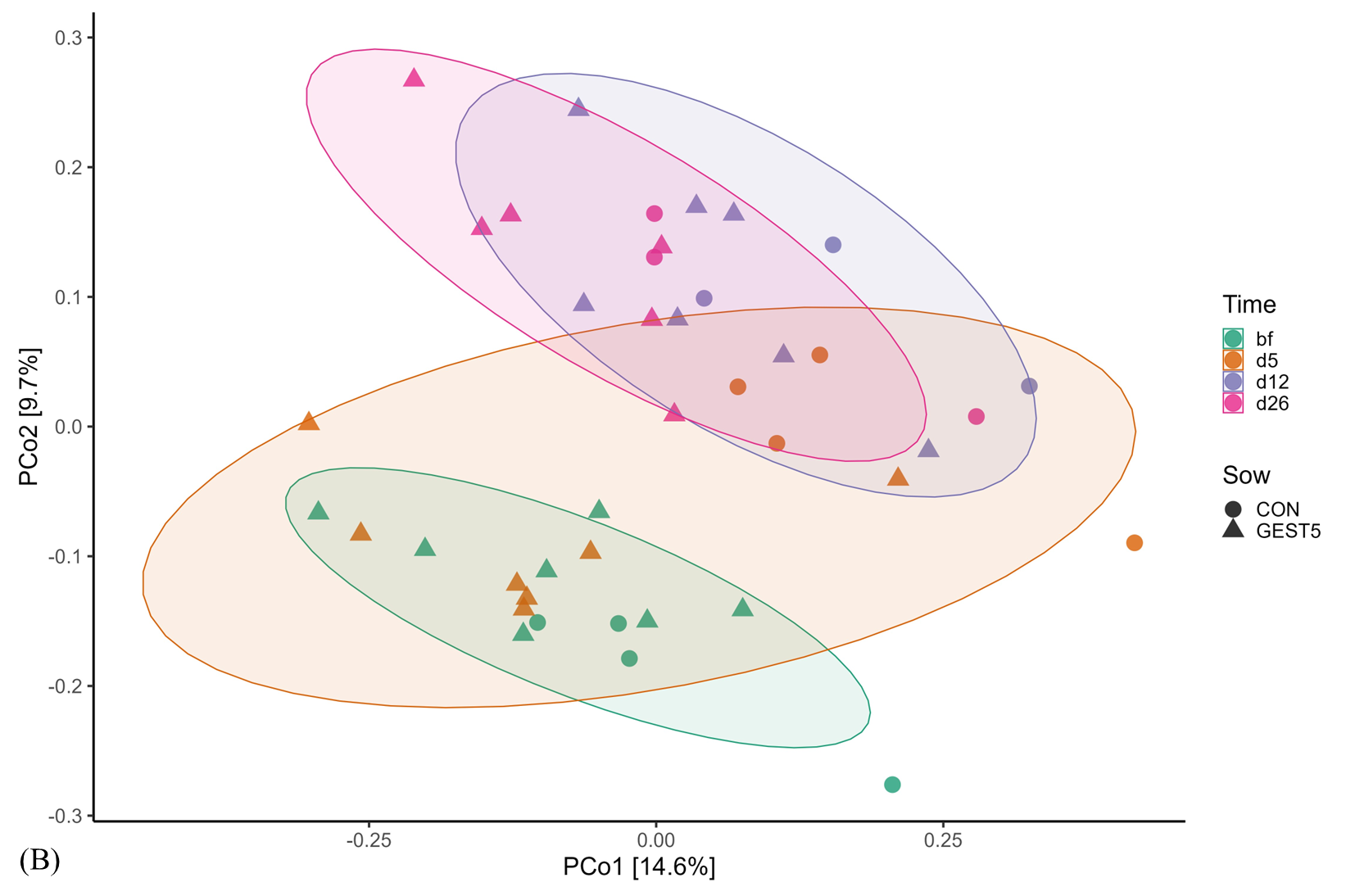


Supplementary Fig. S1. Effect of sow feeding treatment (CON or GEST5) on alpha and beta diversity of the microbiota in the sow faecal samples collected on day 109 of gestation (bf) and on day (d) 5, 12 and 26 after farrowing on the Irish research farm, where CON = sows fed with a lactation diet from one day post-farrowing until weaning; GEST5 = sows fed with a gestation diet for 5 days post-farrowing followed by a lactation diet until weaning. (A) Alpha diversity indices (Observed, Shannon and Inverse-Simpson) in the sow faecal samples across all sampling time points. (B) PCoA plot (β-diversity) of the faecal microbiota of sows across all sampling time points. Shapes indicate the sow feeding treatments and colours indicate the faecal sample collection time points.


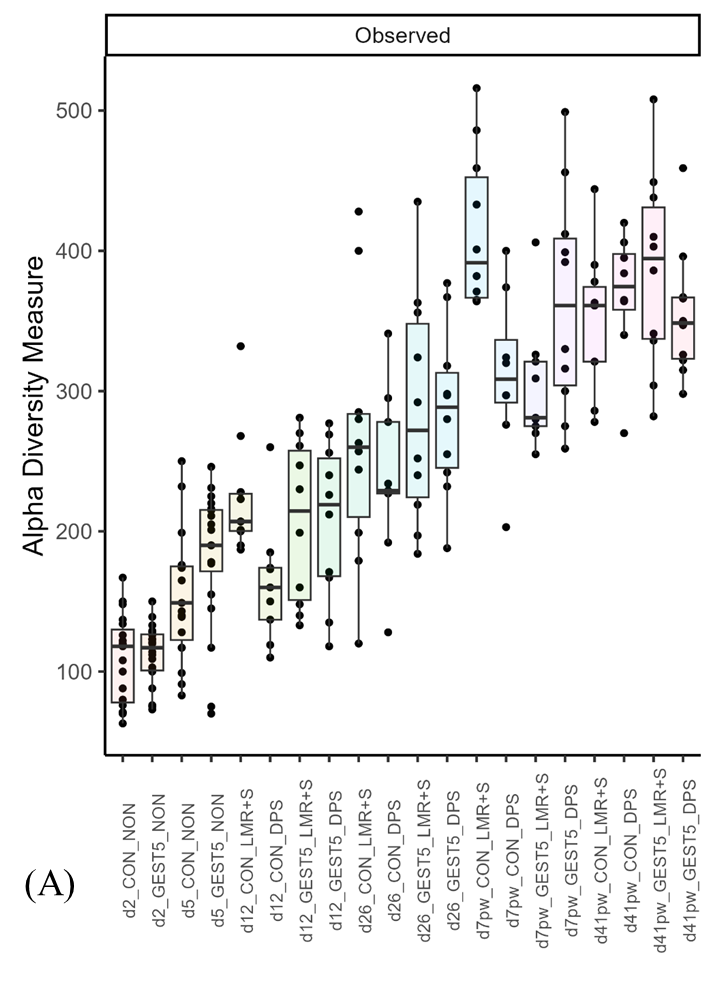

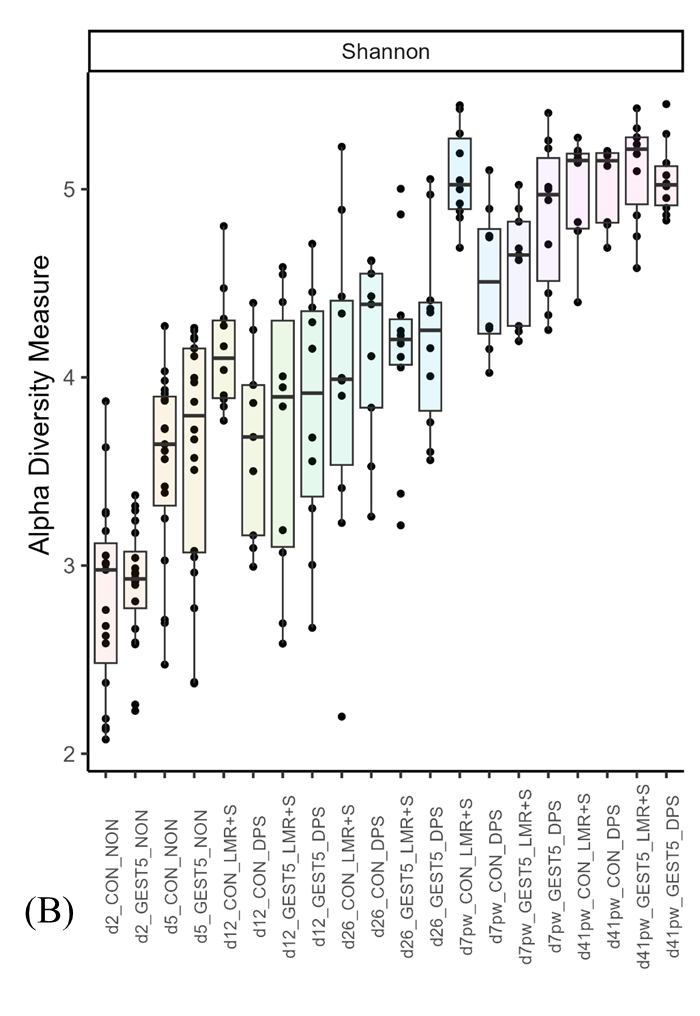


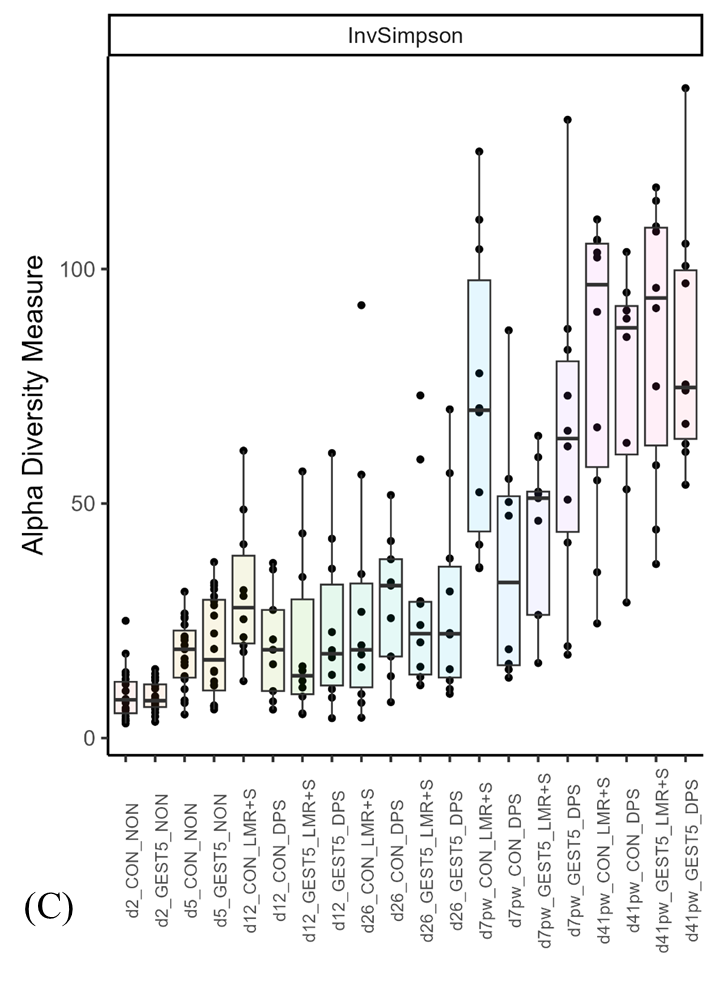


Supplementary Fig. S2. Effect of sow feeding treatment (CON or GEST5) and creep feeding treatment (DPS or LMR+S) on the alpha diversity indicators (A=Observed, B=Shannon and C=Inverse-Simpson indices) in piglet faecal samples collected on day (d) 2, 5, 12 and 26 after birth and day 7 (d7pw) and 41 post-weaning (d41pw) on the Irish research farm. CON = sows fed with a lactation diet from one day post-farrowing until weaning; GEST5 = sows fed with a gestation diet for 5 days post-farrowing followed by a lactation diet until weaning; DPS = suckling piglets provided with dry pelleted starter diet from day 5 to weaning; LMR+S = suckling piglets provided with a liquid mixture of milk replacer and starter diet from day 5 to weaning; NON = no creep feeding treatment.


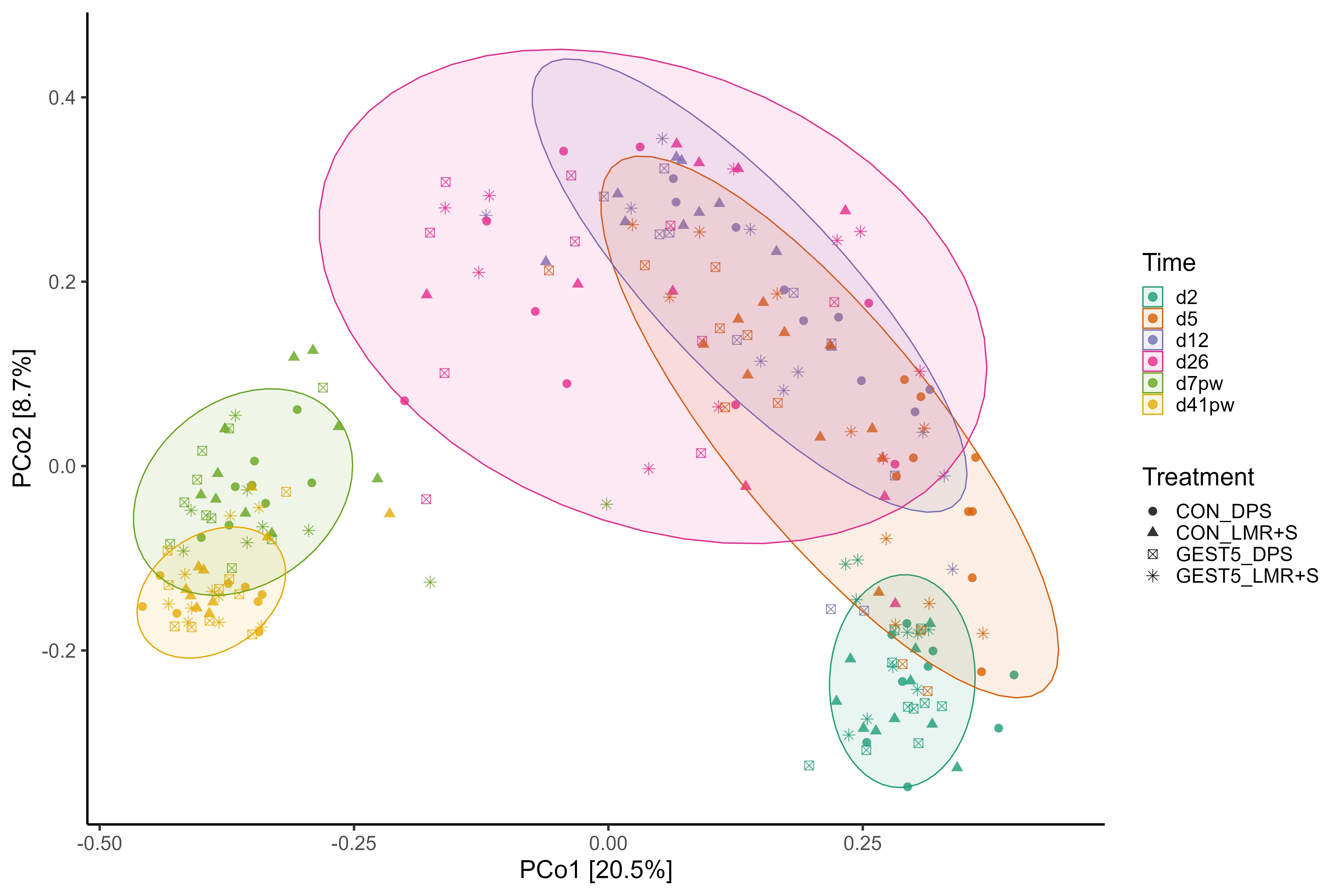


Supplementary Fig. S3. PCoA plot (β-diversity) of the faecal microbiota of pigs from all four treatments across all sampling time points. Colours indicate the faecal samples collected at day (d) 2, 5, 12 and 26 after birth and day 7 (d7pw) and 41 post-weaning (d41pw) on the Irish research farm. Shapes indicate the treatments as follows: CON = Sows fed with a lactation diet from one day post-farrowing until weaning; GEST5 = Sows fed with a gestation diet for 5 days post-farrowing followed by a lactation diet until weaning; DPS = Suckling piglets provided with dry pelleted starter diet from day 5 to weaning; LMR+S = Suckling piglets provided with a liquid mixture of milk replacer and starter diet from day 5 to weaning.
